# PD-1 blockade does not enhance alloimmunization after allogeneic dendritic cell vaccination in cancer patients

**DOI:** 10.3389/fimmu.2026.1763434

**Published:** 2026-03-04

**Authors:** Severine Planel, Guillaume Vayssière, Gianni Maggipinto, Estelle Leplus, Karine Laulagnier, Florence Renard, Francoise Myster, Marie Gerard, Ingel Demedts, Kristof Cuppens, Elvire Pons-Tostivint, Els Wauters, Frank J. Borm, Anne Sibille, Benoît Colinet, Maurice Pérol, Willemijn S. M. E. Theelen, Bonne Biesma, Charlotte Van De Kerkhove, Eva Buchmeier, Friederike C. Althoff, Sofie Derijcke, Denis Moro-Sibilot, Frédérique Cantero, Laurence Chaperot, Philippe Saas, Marcin Skrzypski, Johan Vansteenkiste, Joël Plumas

**Affiliations:** 1 R&D Department, PDC*line Pharma France, Grenoble, France; 2 G.M. Consultant Company, Liège, Belgium; 3 R&D and Clinical Departments, PDC*line Pharma Belgique, Liège, Belgium; 4 Human Leukocyte Antigen (HLA) Laboratory, Etablissement Français du Sang (EFS) Rhone-Alpes Auvergne, Grenoble, France; 5 Pulmonary Diseases, AZ Delta Campus Rumbeke, Roeselare, Belgium; 6 Pulmonology and Thoracic Oncology Department, Jessa Hospital, Hasselt, Belgium; 7 Medical Oncology Department, Nantes University Hospital, Nantes, France; 8 Respiratory Oncology Unit (Pulmonology), University Hospitals Leuven, Leuven, Belgium; 9 Department of Pulmonology, Leiden University Medical Center, Leiden, Netherlands; 10 Department of Pulmonology, Centre Hospitalier Universitaire de Liège, Liège, Belgium; 11 Pneumology Department, Grand Hôpital de Charleroi, Site Notre Dame, Charleroi, Belgium; 12 Medical Oncology Department, Centre Léon Bérard, Lyon, France; 13 Thoracic Oncology Department, Netherlands Cancer Institute, Amsterdam, Netherlands; 14 Pulmonology, Jeroen Bosch Hospital, ‘s-Hertogenbosch, Netherlands; 15 Clinical Study Centre, Vitaz campus Sint-Niklaas Moerland, Sint-Niklaas, Belgium; 16 Hematology and Oncology Department, Kliniken der Stadt Koln gGmbH, Köln, Germany; 17 Hemato-Oncology Department, Universitätsklinikum Frankfurt (Johannes-Wolfgang Goethe-Universität), Frankfurt Am Main, Germany; 18 Pulmonology: Thoracic Oncology, AZ Groeninge, Campus Kennedylaan, Kortrijk, Belgium; 19 Thoracic Oncology, Service d'Hospitalo-Universitaire de Pneumologie et Pneumo-Oncologie (SHUPP), Centre Hospitalier Universitaire (CHU) Grenoble-Alpes, Grenoble, France; 20 Université Grenoble Alpes, Inserm U 1209, CNRS UMR 5309, Institute for Advanced Biosciences (IAB), team Translational Immunology and Immunotherapy in Chronic Disease, Grenoble, France; 21 Recherche et Développement, Etablissement Français du Sang Auvergne-Rhône-Alpes, Grenoble, France; 22 Department of Oncology and Radiotherapy, Medical University of Gdańsk, Gdańsk, Poland

**Keywords:** cancer vaccine, clinical trial, translational research, allogeneic humoral response, immune-checkpoint point blockade, immunotherapy, plasmacytoid dendritic cells

## Abstract

**Background:**

Blocking programmed cell death protein 1 (PD-1) has become a standard cancer immunotherapy, increasingly used in kidney, liver, or heart transplant recipients who develop skin cancer or hepatocellular carcinoma, despite the increased risk of graft failure or rejection. The mechanism of action of PD-1 blockade relies on stimulating CD8+ T cell activity, but its impact on humoral immunity in general and on alloimmunization in particular remains uncertain.

**Objective:**

The aim of this study was to investigate the impact on anti-PD-1 treatment on alloimmunization.

**Methods:**

The effect of anti-PD-1 treatment on the generation of anti-HLA (Human Leucocyte Antigen) antibodies was investigated in 72 patients with non-small cell lung cancer vaccinated with an allogeneic plasmacytoid dendritic cell line (PDC*line; six weekly injections), with or without pembrolizumab administered every three weeks. The kinetics and functionality of the anti-HLA generated were analyzed.

**Results:**

The results show that 51.4% of the patients developed anti-HLA antibodies, primarily dependent on the vaccine dose. In 60% of cases, the antibody response appeared after the sixth injection, peaked after one month, and then gradually declined over two years. Anti-HLA class II antibodies appeared earlier than class I antibodies. Functional assays demonstrated complement-dependent cytotoxicity against allogeneic B lymphocytes and PDC*line cells in the serum of some patients, with no difference related to treatment. PD-1 blockade did not alter the magnitude, kinetics, or cytotoxic potential of the vaccine-induced humoral response.

**Conclusion:**

These results indicate that, during allogeneic human vaccination, PD-1 signaling exerts a limited effect on antibody production and effector function, suggesting a more complex regulatory role in humoral immunity than previously thought.

## Introduction

Blocking the PD -1 (programmed cell death protein-1) receptor has been widely used since 2012 to treat a growing number of cancer indications (1). Due to their crucial function in anti-tumor CD8+ (Cluster of Differentiation) T-cell responses, therapeutic antagonist antibodies targeting PD-1 are now used as first-line monotherapy in advanced stages of melanoma, non-small cell lung cancer (NSCLC), and tumors with high microsatellite instability or mismatch repair deficiency (2).

Because cancer is a frequent complication after solid organ transplantation, treatments targeting the PD-1/PD-L1 axis are increasingly used in kidney, liver, or heart transplant recipients who develop skin cancer or hepatocellular carcinoma, despite the increased risk of graft failure or rejection (3–5). Rejection mechanisms appear to involve T-cell-mediated rejection or mixed T-cell and humoral-mediated rejection (6, 7). To date, no conclusive evidence has been found regarding the modulation of donor-specific antibodies following PD-1 blockade.

It has been shown that blocking the interaction between PD-1 expressed on activated CD8+ T cells and its ligands PD-L1/L2 expressed on antigen-presenting cells or tumor cells can reverse T-cell exhaustion, promote their proliferation, enhance their cytotoxic functions, and inhibit their apoptosis (8, 9) thereby providing real clinical benefit to patients. PD-1 is also highly expressed on T follicular helper cells (Tfh) (10–12), which closely orchestrate the differentiation and maturation of B lymphocytes in the germinal centers of lymphoid organs, which ultimately transform into plasma cells generating long-lasting antibody responses.

In experimental models, it has been shown that PD-1 signaling impairs Tfh function and antibody production (13, 14). Conversely, PD-1 blockade leads to Tfh accumulation (14–17) and enhances specific humoral response (14, 16, 17).

In humans, the effect of anti-PD1 blockade studied in patients with different cancers showed an increase of proliferative activity of circulating Tfh and B cells (18) without however altering the subtypes of circulating B cells (19). In patients undergoing anti-PD-1 treatment, single-dose influenza or SARS-Cov2 (Severe acute respiratory syndrome coronavirus 2) vaccines does not appear to have a significant effect on antiviral antibody levels (18, 20, 21), but they do affect the characteristics of circulating Tfh cells (18).

Therefore, the effects of PD-1 blockade on the onset of the humoral immune response in humans remain poorly understood. In order to provide new data on the impact of anti-PD-1 treatment on humoral responses, we have exploited a unique clinical setting that provided a rational model in which the same allogeneic cellular vaccine was administered with or without anti-PD-1 antibodies to 72 cancer patients.

We studied the generation of antibodies over time against allogeneic HLA molecules in patients with NSCLC treated with the allogeneic cell-based cancer vaccine PDC*lung01, with or without pembrolizumab (22) (NCT03970746). PDC*lung01 consists of an HLA-A*02:01-positive human plasmacytoid dendritic cell line (PDC*line) loaded with peptides. The PDC*line cells are simply irradiated without being activated or matured. The mechanism of action of PDC*line cells is based on the direct activation of antigen-specific CD8+ T cells, which we have demonstrated *in vitro* (23, 24), in humanized immunodeficient mice (25), and in humans in two clinical trials conducted in patients with melanoma (26) and lung cancer (22) as mentioned above.

In the PDC*lung01 clinical trial, the vaccine was injected six times alone or in combination with pembrolizumab standard-of-care treatment starting at the same time. Since patients were selected only for their HLA-A*02:01 matching with the PDC*line, the emergence of antibodies against other HLA class I and class II molecules has been carefully evaluated and the allogeneic humoral response was regularly monitored for two years. The impact of PD-1 blockade was examined on the initiation and kinetics of anti-HLA antibody generation, targeted antigens, and functionality of the antibodies generated. Contrary to expectations, no enhancement of humoral response was observed in patients treated with PDC*lung01 in combination with pembrolizumab compared to patients treated with PDC*lung01 alone. These results indicate that anti-PD1 treatment does not have a great impact on alloimmunization in humans.

## Materials and methods

### PDC*line cells and PDC*lung01 product

PDC*line cells were grown in cell suspension in X-VIVO 15 serum-free medium (Lonza, Walkersville, Maryland, USA) in the presence of 10µg/mL gentamycine (Invitrogen, Thermo Fisher Scientific, Waltham, Massachusetts, USA) at 37 °C, 5% CO2, without addition of any growth, differentiation or maturation factors as previously described (26). Their HLA typing was done by next-gene sequencing (NGS) and was the following: HLA-A*02:01, HLA-B*07:02, HLA-B*44:02, HLA-C*05:01, HLA-C07:02, HLA-DRB1*01:03, HLA-DRB1*08:01, HLA-DQB1*04:02, HLA-DQB1*05:01, HLA-DPB1*02:01 and HLA-DPB1*04:01.

PDC*lung01 is a therapeutic vaccine comprising irradiated PDC*line cells loaded with 7 tumor-associated antigenic peptides. Peptides were loaded individually on batches of the cell line that were mixed together to obtained the final drug product (DP). The following HLA A*02:01 restricted peptides were used, derived from six NSCLC antigens i.e. NY-ESO-1, MAGE-A3, MAGE-A4, Multi-MAGE, MUC1, Survivin, and from Melan-A melanoma antigen chosen as positive control. The vaccine was provided in ready-to-use aliquots containing the frozen PDC*lung01 cell suspension (22).

### Study design

**Figure 1:**
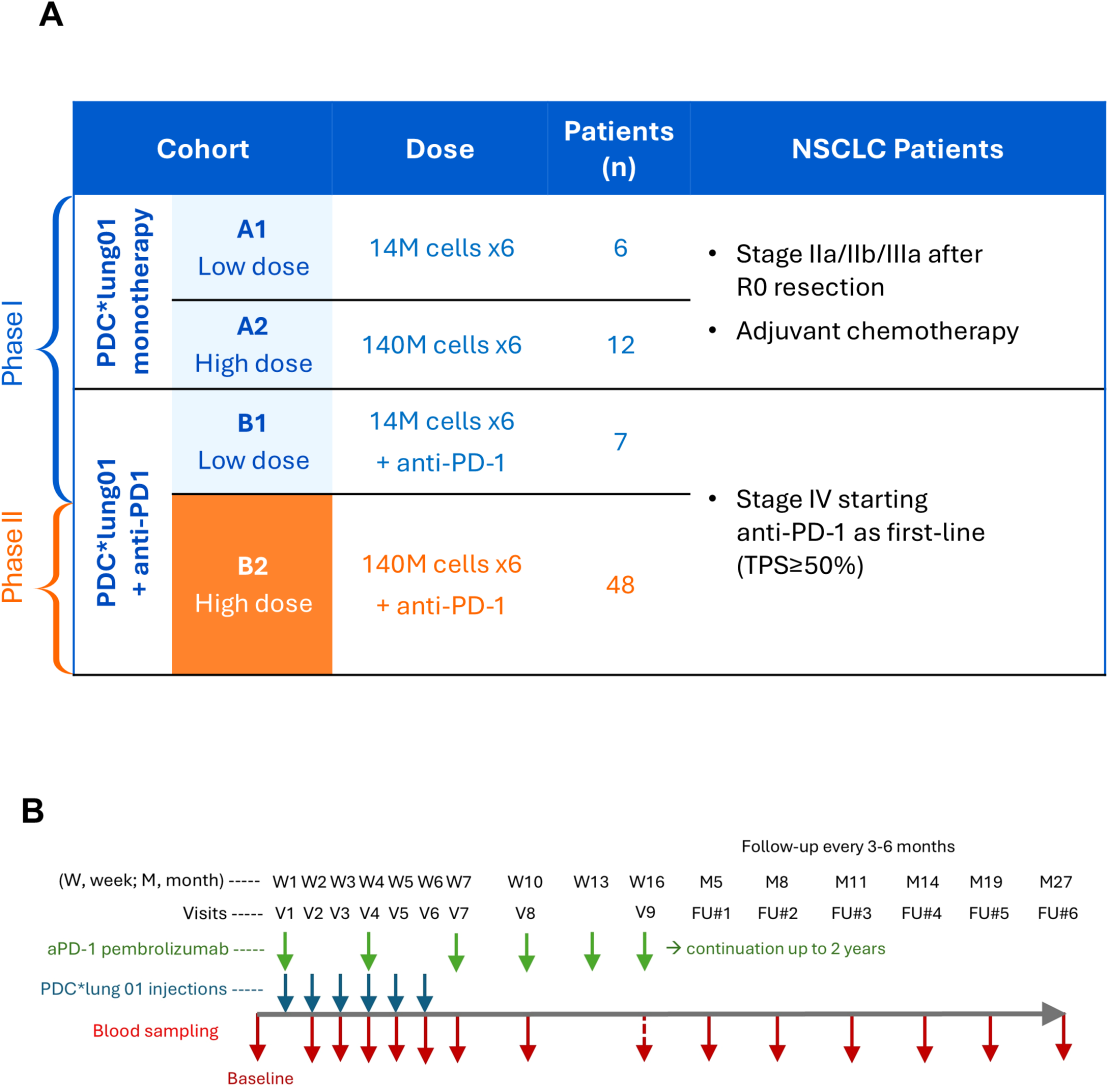
PDC-LUNG-01 clinical trial design. **(A)** Description of the four patient cohorts: the number of injected cells per visit, the number of patients and the inclusion criteria. **(B)** Timeline of treatments and blood sampling. W, week; M, Month; V, visit; FU, Follow-up visit.

Overall, 73 patients were enrolled in that study and divided into 4 Cohorts: 6 in A1 (low dose DP), 12 in A2 (high dose DP), 7 in B1 (low dose DP with anti-PD-1), and 48 in B2 (high dose DP with anti-PD-1) (**Figure 1A**, **Supplementary Table 1**). Patients received 6 weekly injections of PDC*lung01, either at low doses (14 million cells, cohorts A1 and B1) or high doses (140 million cells, cohorts A2 and B2), each dose was split to inject half of the cells intravenously and half subcutaneously.

Anti-PD-1 (pembrolizumab) was administered IV every three weeks until progression or up to 2 years. Since PDC*line cells express the HLA-A*02:01 molecule, only patients with documented HLA-A*02:01 expression and absence of antibodies against the HLA molecules expressed by PDC*line cells were eligible. Patient demographics are presented in **Supplementary Table 1**. Of note, 2 patients of Cohort A2, 1 patient of Cohort B1, and 6 patients of Cohort B2 received only 1 to 5 PDC*lung01 injections. Except one patient in Cohort B2, all patients were included in anti-HLA antibody (Ab) detection analyses since they all had at least one injection of the vaccine and one assessable visit. As detailed in **Figure 1B**, the presence of anti-HLA Abs was evaluated at the screening (baseline) visit (V), then from V2 to V5, at V7, and V8. In case of positivity up to V8, the evaluation of Abs was pursued until their complete disappearance at the follow-up (FU) visits. Moreover, an additional blood volume was collected at V6 in case of positivity between V2 and V5, or at FU#1, FU#2 or FU#3 when positive later and was specifically dedicated to the functionality study of Abs (flow cytometry cross-match and cytotoxicity). The complement-dependent cytotoxicity (CDC) kinetic study was carried out with samples collected at earlier or later timepoints when patients were positive for anti-HLA Ab detection and if additional sampling was available.

### Detection of anti-HLA antibodies

For detection of anti-HLA antibodies, sera for all patients at all timepoint available were used (A1: n=6; A2: n=12; B1: n=7, B2; n=47). Patient’s sera were analyzed for anti-HLA IgG antibodies with the LIFECODES Single Antigen assay (Immucor/Werfen) using Luminex™ technology per manufacturer protocols (27). Results were expressed as mean fluorescence intensity (MFI) and interpreted using MatchIT! Antibody Software.

### Healthy donors: cells and sera

Blood from healthy donors (HDs) was obtained through Etablissement Français du Sang (Grenoble, France) under informed consent (biological collection DC-2016-2815, French Blood Bank). PBMCs were isolated by centrifugation density gradient with lymphocyte separation medium (Eurobio Scientific), and frozen at −150 °C in the presence of Dimethyl Sulfoxyde (Sigma Aldrich), and used for B-cell purification (EasySep™ Human B Cell Enrichment Kit, StemCell). Purity exceeded 95% by CD3/CD19 staining (BD Bioscience).

Pooled sera from eight anonymized HDs provided complement for CDC assays or irrelevant immunoglobulins for flow cytometry cross-match assays. Sera were pooled only if clear, heat-inactivated at 56 °C (30 min) to generate decomplemented fractions (dHS), and stored at −80 °C until use.

B cells from selected HDs with relevant HLA profiles were used to match or not target antigens recognized by patient antibodies (**Supplementary Table 2**). Typing was performed via NGS, except HD#02 (sequence-specific oligonucleotides).

### Quantification of surface molecules

Expression of membrane complement-regulatory proteins (mCRPs) and HLA molecules on PDC*line cells, B cells, and monocytes was quantified using QIFIKIT (Agilent DAKO) (28, 29). The primary mouse antibodies were the following: CD46 (clone EA.3), HLA-DQ (clone Tü169), HLA-DR/DP/DQ (clone Tü39), HLA-A2 (clone BB7.2), IgG1 (clone MP/OPC-21), IgG2a (clone G155-178), and IgG2b (clone 27-35) were from BD Biosciences; CD55 (clone JS11KSC2.3) and CD59 (clone P282E) were from Beckman Coulter; HLA-B7 (clone BB7.1) and HLA-ABC (clone W6/32) were from Biolegend; HLA-DP (clone BRAF B6) was from Santa Cruz; and HLA-DRB1 (clone HLA-DRB/1067) was from Abcam. The secondary antibody used was a FITC-polyclonal goat anti-mouse from Agilent (ref F0479). For PBMCs, AF647-conjugated anti-CD20 (Bio-Techne) identified B cells. Monocytes were gated by morphology (SSC-A vs FSC-A). Median fluorescence values were converted to Antibody-Binding Capacity (ABC) using the QIFIKIT calibration beads. Specific ABC (“antigen density”) was calculated after isotype correction.

### Flow cytometry cross-match assay

To measure antibody binding, PDC*line or control B cells were incubated with decomplemented patients or controls sera. Eleven A2 and sixteen B2 patients positive for anti-HLA antibodies were analyzed.

Cells (1×10^5^) were incubated for 20 min at 4 °C with patients’ sera, dHS, or PBS, then washed in PBS–2% heat-inactivated fetal bovine serum (FBS, Gibco), and stained with FITC-goat anti-IgG secondary antibody (Jackson ImmunoResearch Labs Cat# 109-095-098, RRID: AB_2337658) for 15 min. Following washing and fixation with FACS Lysing solution (BD Bioscience), fluorescence was acquired on FACS Canto II (BD Bioscience) with Diva v9.1.0 and analyzed with FlowJo v10.10.

### Complement-dependent cytotoxicity assay

To assess Complement-Dependent Cytotoxicity (CDC), 25,000 to 100,000 PDC*line or control B cells were incubated with patient serum or control anti-HLA antibodies (anti-pan Class I, ref 824101 from Bio-Rad; anti-pan Class II, ref hla-c2 from Invivogen) in the presence of functional complement (from healthy donors serum pool or patient serum) for 1 h at 37 °C. Cell death was identified by 7-AAD staining (ref 559925 from BD Bioscience) and quantified by flow cytometry (30).

Negative controls used decomplemented serum. CDC positivity was defined as >20% cell death and ≥2× increase vs control. For the CDC experiments, only sera from patients containing anti-HLA antibodies and with validated complement functionality were included. This criterion was met for all sera specifically collected for functional analysis (A2, n = 10; B2, n = 16), but not for all sera collected for anti-HLA determination (see “Patients’ complement validation” in Supplementary Figures). Serum complement functionality was validated using control anti-pan Class II antibodies with B cells from HD#01–03, requiring ≥24.5% cytotoxicity (half the median across 43 validations).

To evaluate mCRPs functionality, PDC*line cells were preincubated with antibodies blocking CD46 (Thermo Fisher Scientific Cat# MA1-40183, RRID: AB_1072487), CD55 (clone BRIC 2016 from Bio-Rad), or CD59 (clone YTH53.1Sigma-Aldrich) at 5 µg/mL for 15 min before CDC assays (31). Serum complement activity was verified using PDC*line cells, with ≥20% cytotoxicity set as assay validity threshold (half the median of 58 assays).

### Statistical analysis

One-way ANOVA with multiple comparisons or t-test were used for comparison conditions. Chi^2^ test was used to compare the immunogenicity against HLA Class I and HLA Class II in the whole population of patients. Data were analyzed using PRISM software (v10).

## Results

### Anti-HLA antibodies in patients treated with PDC*lung01 with or without anti-PD-1

**Figure 2:**
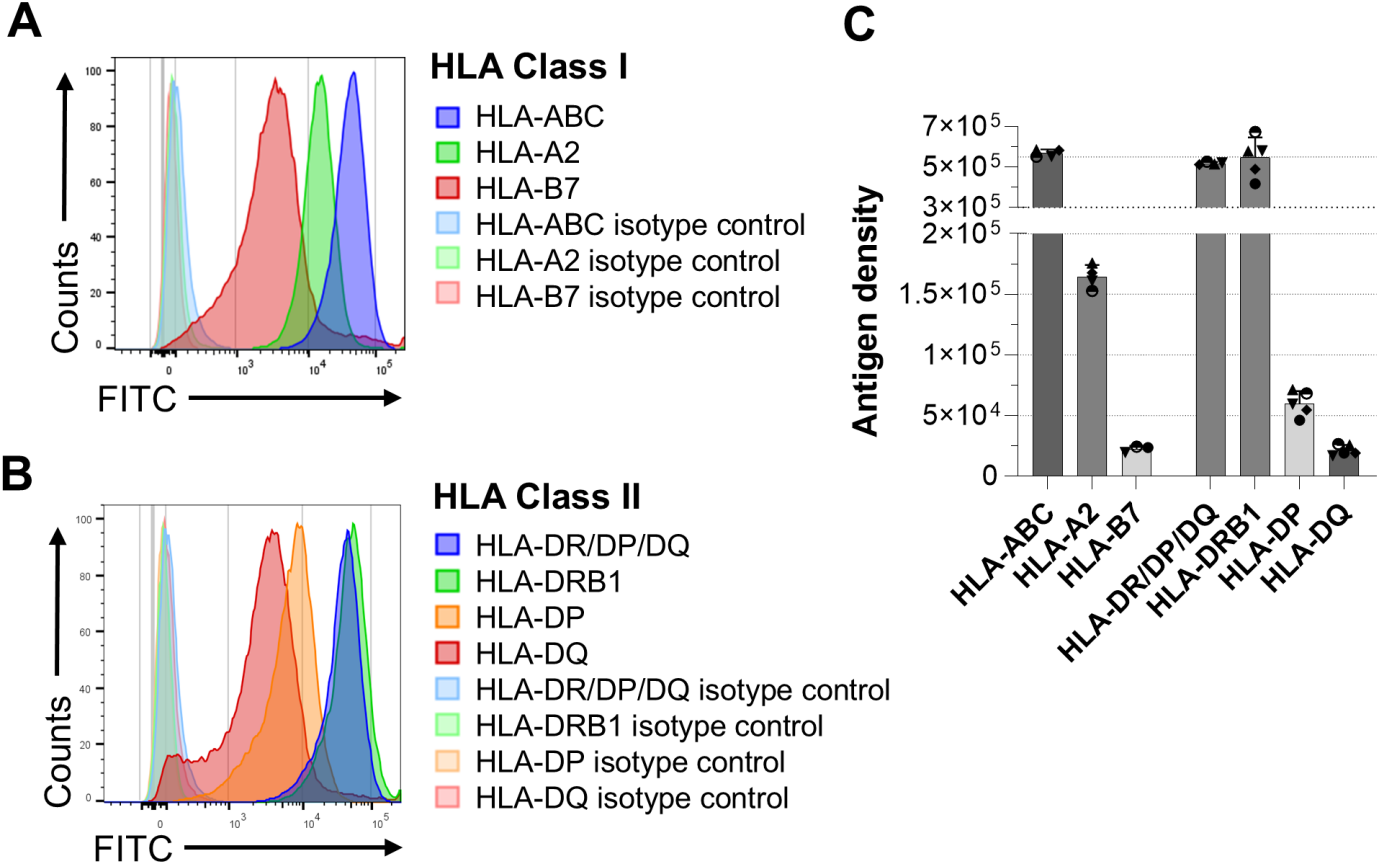
Expression of HLA Class I and Class II molecules by PDC*line cells. HLA Class I (HLA-ABC, HLA-A2, HLA-B7) and Class II molecules (HLA-DR/DP/DQ, HLA-DRB1, HLA-DP, HLA-DQ) were quantified on PDC*line cells. **(A, B)** Representative histograms showing PDC*line cell labelling with FITC-conjugated anti-HLA Class I **(A)** and -HLA Class II **(B)** antibodies. Antibodies specific to cognate isotypes were used as control. **(C)** The means +/- SD of 3 to 5 experiments measuring the HLA antigen density at the PDC*line cell surface are shown.

The evaluation of anti-PD-1 effect on anti HLA-antibodies generation was evaluated in the 4 cohorts treated with PDC*Lung01, representing a homogeneous population of patients (**Figure 1B**). PDC*line cells express high levels of HLA class I and class II molecules, with HLA-ABC and HLA-DR/DP/DQ antigen density reaching 5x10^5^ molecules per cell much higher than that observed on B cells or monocytes (**Figure 2**, **Supplementary Figure 1**).

As expected, the level of incompatibility for HLA-B/C/DR/DP/DQ molecules between PDC*lung01 and patients, as assessed by the number of mismatches per patient (mean = 6, **Supplementary Figure 2**) was high and equivalent in patients regardless of treatment. In total, 51.4% of patients developed alloantibodies, among whom only 7.7% (1/13) of patients treated with low dose PDC*lung01 (**Supplementary Figure 3**), while 59.3% (35/59) of patients treated with high dose were tested positive for anti-HLA antibodies, clearly indicating an obvious dose effect. Since patients were matched for HLA-A*02:01, no antibodies against this molecule were detected, and only a few patients developed antibodies against HLA-C*05:01 or HLA-C*07:02 (4.6%). In contrast, in patients treated with high dose, HLA alloimmunization appeared more important against class II molecules (59.3%; 35/59) of positive patients than class I molecules (33.9%; 20/59) (**Table 1**; Chi^2^ test, p=0.0056). In addition, among anti-HLA class II molecules, DRB1 induced more immunization than DPB1 and DQB1.

**Table 1:** Generation of anti-HLA^1^ antibodies during PDC*lung01 treatment with or without anti-PD-1^2^.

Cohort/Visit	V2	V3	V4	V5	V7	V8
Anti-HLA Class I (no. (%))*						
Cohort A1**	–	–	–	–	0/6 (0)	1/6 (16.7)
Cohort B1	0/7 (0)	0/7 (0)	0/7 (0)	0/7 (0)	0/7 (0)	0/7 (0)
Cohort A2	0/11 (0)	0/11 (0)	0/11 (0)	2/10 (20)	6/12(50)	6/11 (54.5)
Cohort B2	0/47 (0)	0/46 (0)	1/46 (2.2)	2/46 (4.3)	9/43 (20.9)	12/44 (27.3)
Anti-HLA Class II (no. (%))						
Cohort A1	–	–	–	–	0/6 (0)	1/6 (16.7)
Cohort B1	0/7 (0)	0/7 (0)	0/7 (0)	0/7 (0)	0/7 (0)	0/7 (0)
Cohort A2	0/11 (0)	2/11 (18.2)	5/11 (45.5)	6/11 (63.6)	10/12 (83)	11/11 (100)
Cohort B2	2/47 (4.3)	3/46 (6.5)	5/46 (10.9)	8/46 (17.4)	16/43 (37.2)	24/44 (54.5)

^1^HLA, Human Leucocyte Antigen; ^2^PD-1, Programmed cell death protein 1; *Anti-HLA antibodies were measured in sera collected at injection visits (V) from V2 to V5 plus at V7 and V8. **Sera were not available during the first visits in Cohort A1 and from the first or second visit onwards in the other cohorts.

**Figure 3:**
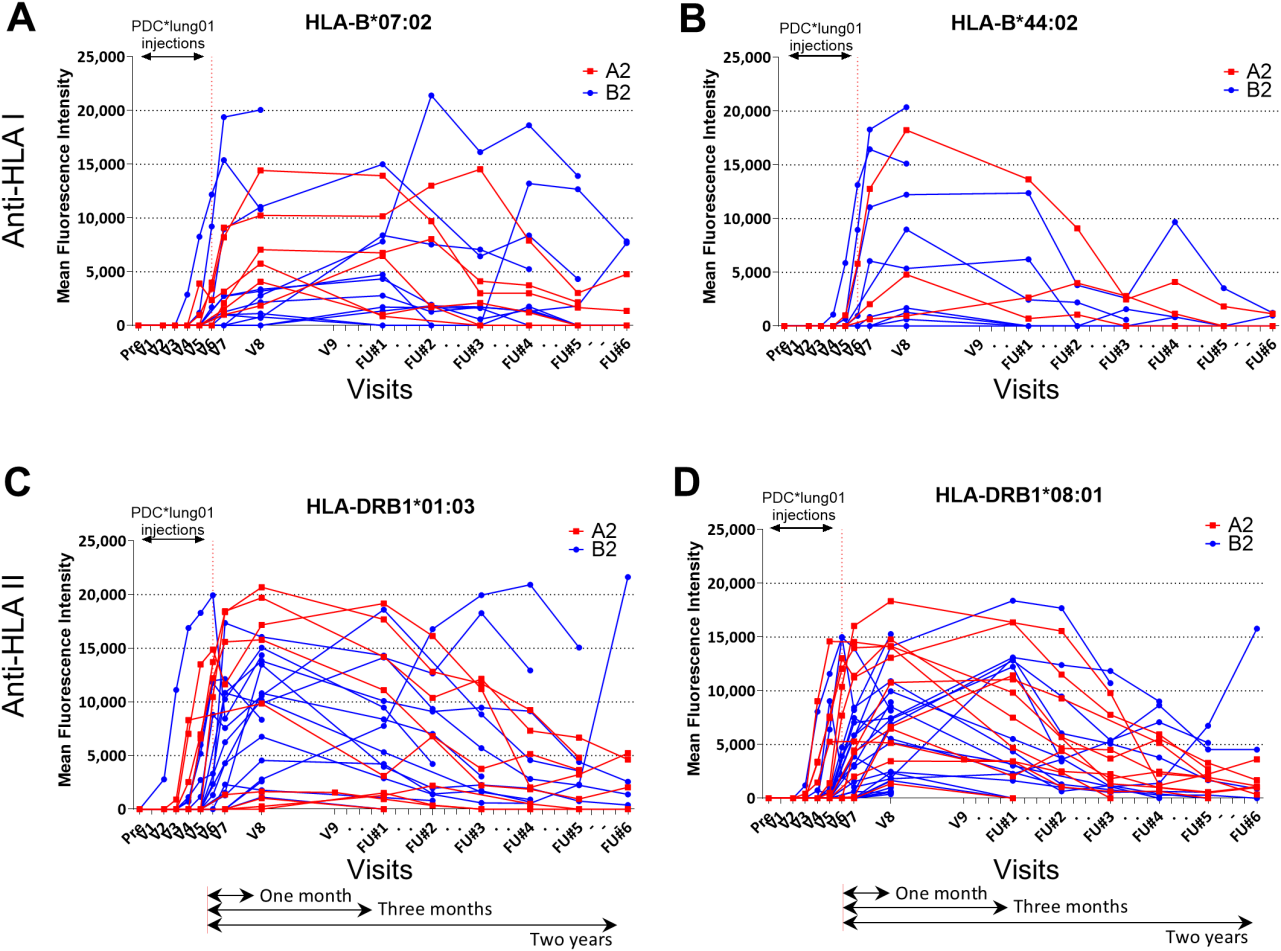
Kinetics of anti-HLA humoral response in patients from cohorts A2 and B2. The detections of anti-HLA Class I **(A, B)** and -HLA Class II **(C, D)** antibodies were performed over time in the sera of patients of cohort A2 (n=12; red) and B2 (n=48; blue). The most represented HLA molecules are shown HLA-B*07:02 **(A)**, HLA-B*44:02 **(B)**, HLA-DRB1*01:03 **(C)**, HLA-DRB1*08:01 **(D)**. V, visit; FU, Follow-Up.

With regard to the intensity and kinetics of anti-class I (HLA-B) and anti-class II (HLA-DRB1) antibodies, very high levels of alloantibodies were observed (>15,000 MFI), generally peaking at V8, one month after the last PDC*lung01 injection, and persisting for approximately six months before decreasing and becoming undetectable within 12 to 24 months depending on patients (**Figure 3**). Similar profiles were obtained for anti-HLA-DQB1 and HLA-DPB1 antibodies (**Supplementary Figure 5**). It is also important to note that anti-class II antibodies appeared as soon as the first or second injection of PDC*lung01 (V2 or V3), while anti-class I antibodies were only detected after V4 or V5 (fourth or fifth injections of PDC*lung01, respectively) (**Table 1**), without being associated with safety issues (22). Patients treated with the combination of PDC*lung01 and anti-PD-1 did not have an earlier anti-HLA response, nor did they show higher levels of anti-HLA antibodies or longer maintenance of these levels compared to patients receiving PDC*lung01 alone (**Supplementary Figure 6**).

### PD-1 blockade does not increase the functionality of patients’ anti-HLA antibodies

The functionality of alloantibodies was studied, particularly their potential for complement-mediated cytotoxicity. We first used methods employed in HLA laboratories to evaluate the overall reactivity of these antibodies by performing cell-based assays using purified B and T cells (lymphocytotoxic assay), completed by detecting C3d deposition (a component of the complement cascade) for some patients. The cells used for lymphocytotoxic assay were selected for their HLA typing (**Supplementary Table 2**) and the two assays were performed in the presence of rabbit complement. The results showed that more than 60% of patients’ sera were positive for C3d deposition and that more than 85% developed complement-dependent cytotoxicity (CDC) mediated by both IgG and IgM immunoglobulins (**Supplementary Table 3**). No increase in these parameters was observed with anti-PD1 treatment.

**Figure 4:**
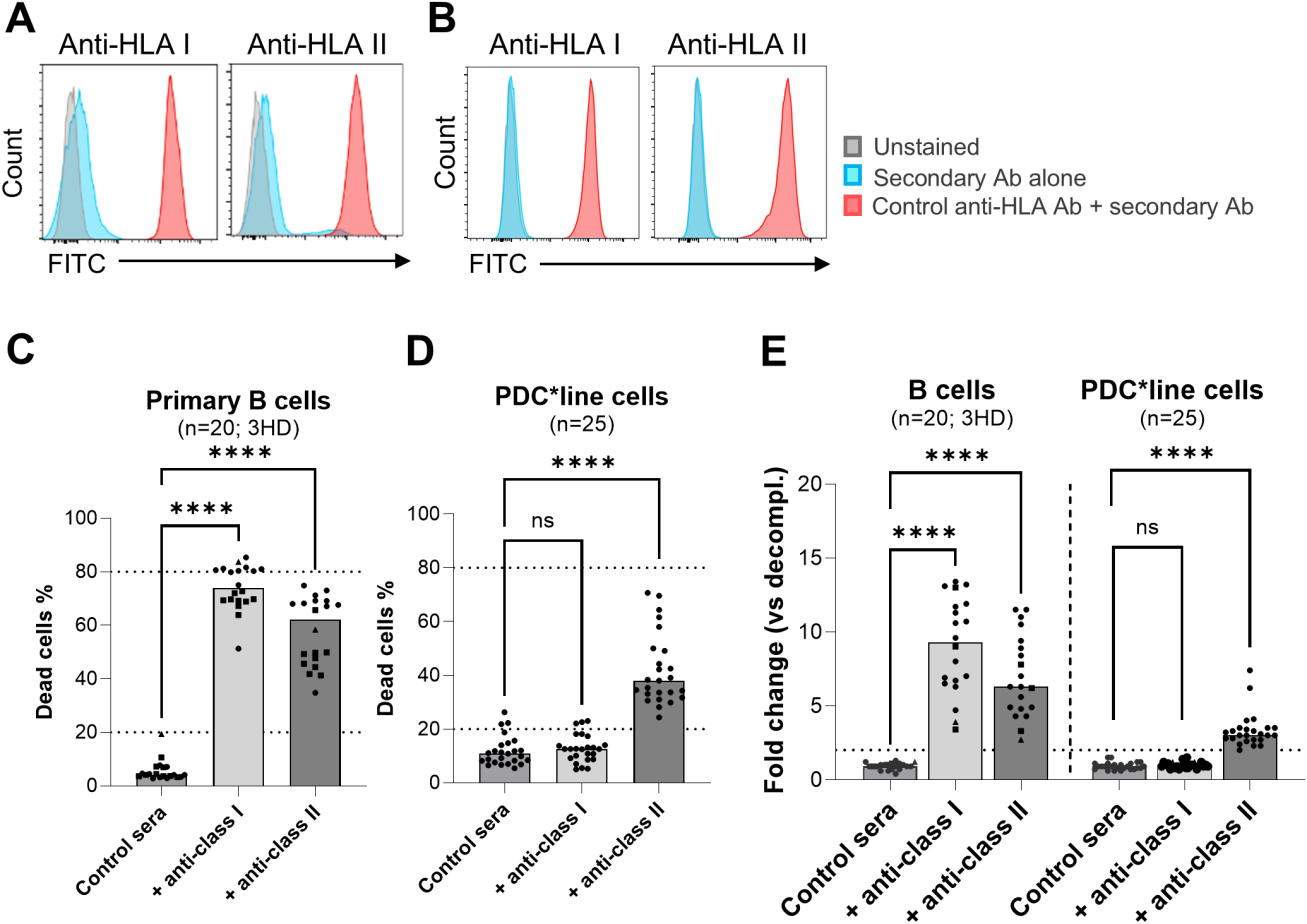
Complement-dependent cytotoxicity (CDC) mediated by control anti-HLA antibodies against control B cells and PDC*line cells. **(A, B)**. Binding of anti-HLA class I and class II control antibodies (red) on primary B cells **(A)** and PDC*line cells **(B)**. Secondary antibody alone (blue) and unstained cells (grey) were used as negative control. **(C, D)** Percentage of B cell and PDC*line cell death after CDC experiments with anti-HLA class I or II control antibodies in the presence of human serum pool as source of functional complement. Human serum pools without anti-HLA control antibodies were used as negative controls (Control sera). In C, the results gathered experiments done with three healthy donors (HD#01-03) of B cells. Decomplemented human sera were used as negative control in each experiment (not shown). The horizontal dotted line indicates the 20% positivity threshold. **(E)** Fold change of the cell death percentage in complete versus decomplemented human donor serum conditions. The horizontal dotted line indicates the twofold positivity threshold. In **(C)** (n=20), **(D)** (n=25), and **(E)**, the bars show the median values. One-way ANOVA with multiple comparisons was used to statistically compare the conditions (control sera, versus +anti-HLA I and +anti-HLA II); ****(p<0.0001), ns (non-significant).

Since the results of these tests do not always correlate with the clinical situation in the field of transplantation (32), we decided to develop a new assay using human sera as source of complement and PDC*line cells or control B cells as target cells. We first validated this test using positive controls for anti-HLA class I or class II antibodies. After verifying the effective binding of these control antibodies to B cells and PDC*line cells (**Figures 4A, B**), cell death was measured after 1 hour of incubation at 37 °C (**Supplementary Figure 7**). As shown in **Figures 4C, E**, a high percentage of cytotoxicity was observed with B cells and the two positive control antibodies. In contrast, with PDC*line cells, moderate CDC was observed only with anti-class II antibodies, suggesting resistance of PDC*line cells to antibody-mediated CDC (**Figures 4D, E**). As PDC*line cells are irradiated within PDC*lung01 vaccine, the resistance of irradiated PDC*line cells to CDC was verified and showed no difference compared to non-irradiated cells (**Supplementary Figure 8**).

We then evaluated the cytotoxic potential of sera from patients treated with PDC*lung01 alone or in combination with anti-PD-1, against PDC*line cells and allogeneic control B cells. Since anti-HLA-DR8 and anti-HLA-DR103 antibodies were highly representative of the alloimmune response of patients, we selected two sources of B cells matched for these HLA molecules. B cells unmatched for these molecules were used as negative controls (**Supplementary Table 2**). First, the complement functionality was validated in patients’ sera from all available samples (**Supplementary Figure 9**). Then, we showed that alloantibodies detected in sera from the timepoints dedicated to the anti-HLA impact study (V6, FU#1, and FU#2; **Figure 1B**) were able to bind to the surface of PDC*line and allogeneic control B cells (**Supplementary Figure 10**) and thus could trigger the cytotoxic cascade induced by the functional complement. No binding was observed to unmatched B cells.

**Figure 5:**
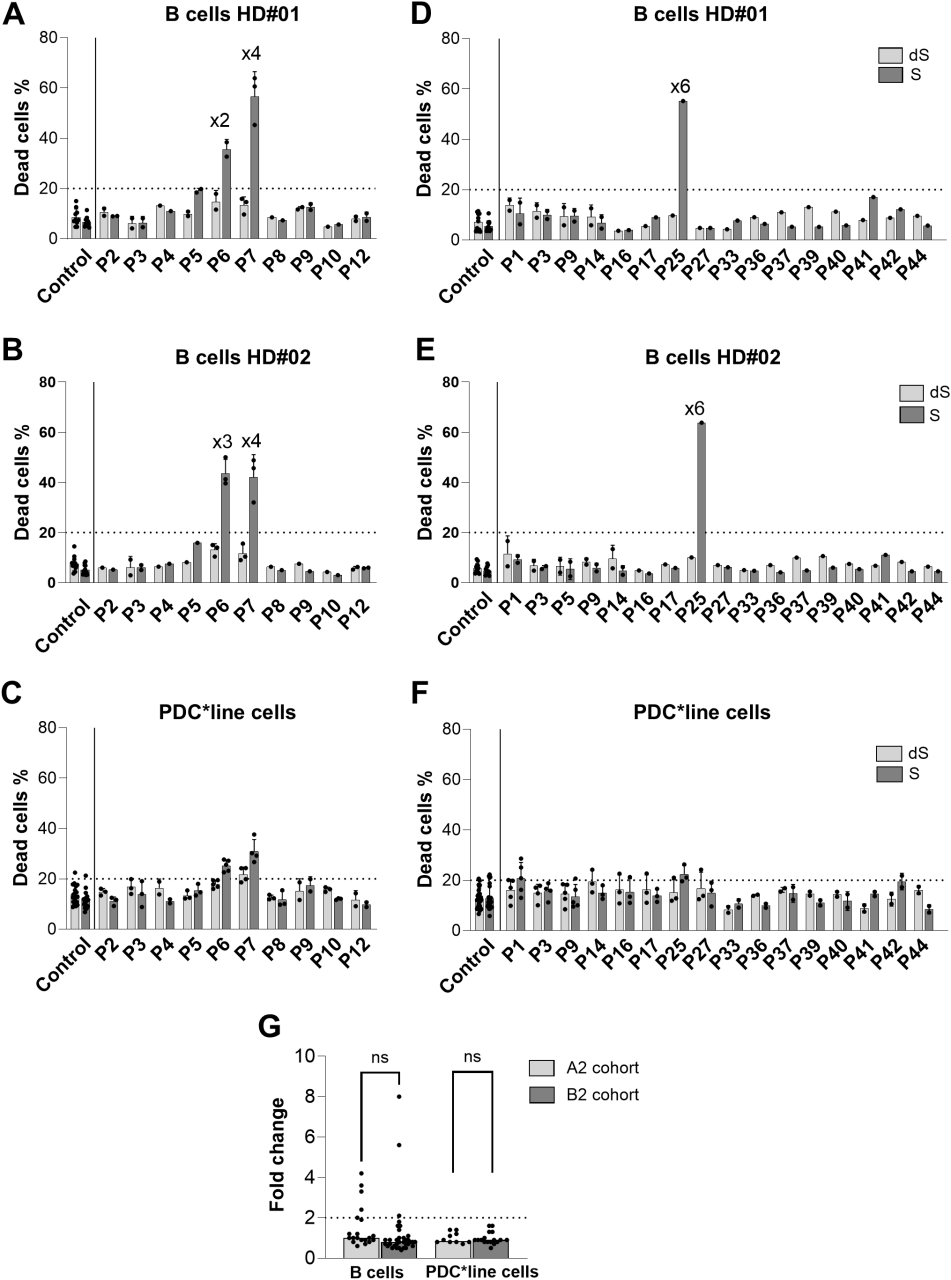
Complement-dependent cytotoxicity (CDC) activity of patients’ sera. CDC activity was measured in sera of patients from cohort A2 [**(A-C)**; n=10] and B2 [**(D-F)**; n=16] against primary B cells from 2 allogeneic healthy donors [HD#01 and HD#02; **(A, B, D, E)** and PDC*line cells **(C, F)**]. The cell death percentage is shown in untreated (S) and decomplemented serum (dS) conditions. The horizontal dotted line indicates the 20% positivity threshold. The “xn” sign above the bar plots indicates the fold change in dead cells between the untreated serum and decomplemented serum conditions for the corresponding patient. Pools of HD sera were used as negative controls (Control sera). The means +SD are presented (n=1–5 for patients’ sera; n=11 to 28 for control sera) depending of targeted cells and experiments. **(G)** The cytotoxicity fold change between S and dS conditions for A2 and B2 patients’ sera is shown. The horizontal dotted line indicates the twofold positivity threshold. The bars show the median values for the 10 and 16 patients of A2 and B2 cohorts, respectively. For B cells, the results with the two allogeneic B cells have been gathered (n=21 for A2; n=35 for B2). t test was used to statistically compare results from A2 and B2 cohort for B cells and PDC*line cells conditions (ns, non-significant).

The CDC potential of the patients’ sera was then evaluated (**Figure 5**). Contrary to what was expected based on the results of the lymphocytotoxicity and C3d assays, only a few sera from both patient cohorts showed antibody-mediated CDC against matched B-cell controls (**Figures 5A, B, D, E, G**). All sera were negative with unmatched control B cells (**Supplementary Figure 11**). Regardless of the treatment received, patients’ sera did not induce any antibody-mediated CDC against PDC*line cells, although they were considered as the best cell target (**Figures 4C, F, G**). Indeed, PDC*line cells were recognized by all alloantibodies, while B-cell controls that were only partially mismatched led to moderate binding of alloantibodies (**Supplementary Figure 10**). Sera from patients treated with anti-PD-1 did not show higher CDC since the results from both cohorts were similar (**Figure 5G**).

**Figure 6:**
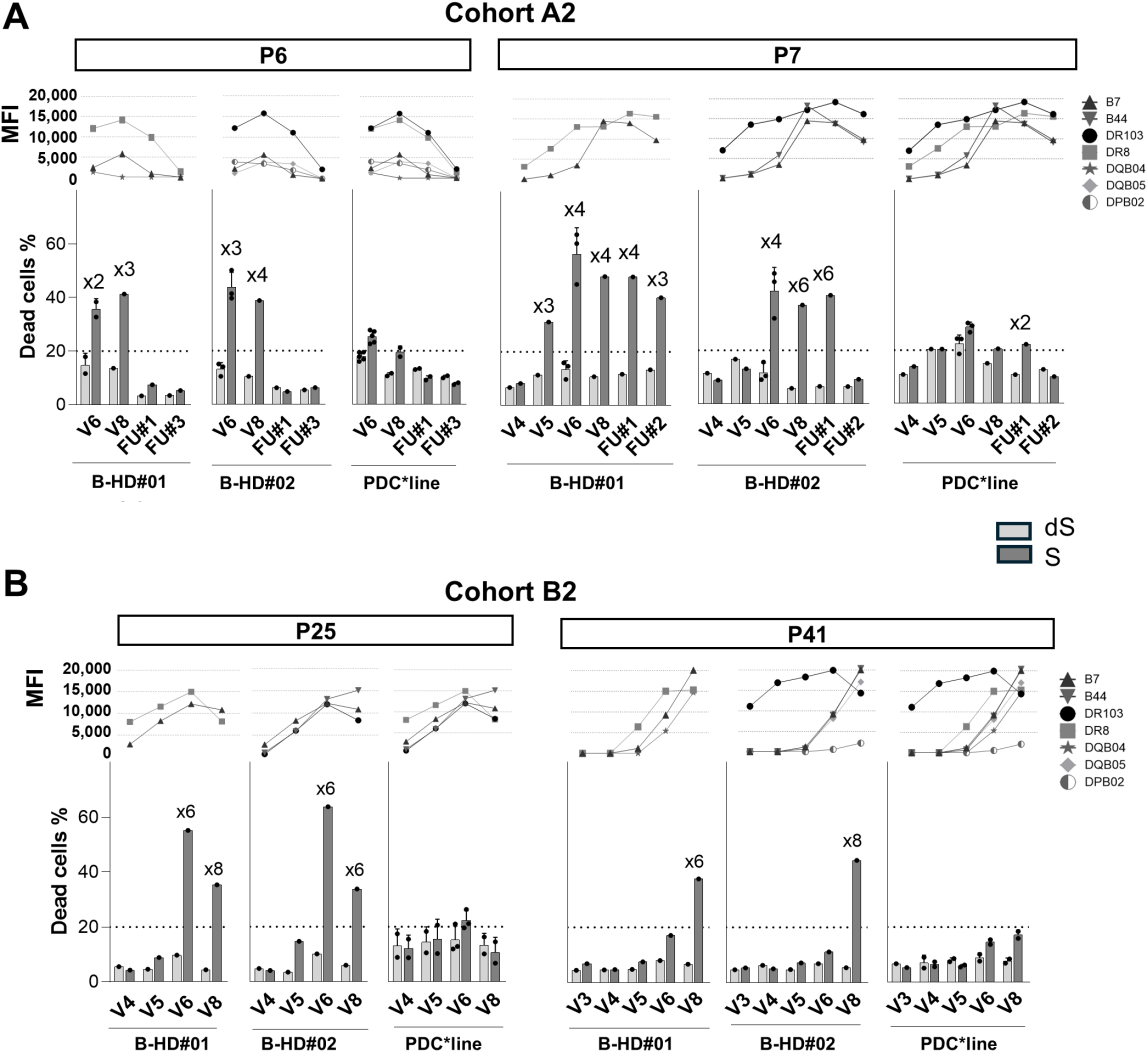
Kinetics of complement-dependent cytotoxicity (CDC) activity according to anti-HLA levels. The sera of two patients (P) from Cohort A2 **(A)** and B2 **(B)** were collected at different timepoints during the treatments (see **Figure 1B**). The line graphs present the mean fluorescence intensity (MFI) of anti-HLA antibodies. The levels of anti-HLA Class I (B7 and B44) and Class II (DR103, DR8, DQB04, DQB05, and DPB02) matching HLA typing of the indicated cell type, are shown. CDC activity was evaluated against primary B cells from two allogeneic healthy donors (HD#01 and HD#02) and PDC*line cells. The bar plots show the cell death percentage in untreated (S) and decomplemented serum (dS) conditions (means +SD; n=1-3). The horizontal dotted line indicates the 20% positivity threshold. The “xn” sign above the bar plots indicates the fold change in dead cells between the untreated serum and decomplemented serum conditions for the corresponding patient.

We then wondered whether sera collected at other timepoints might be positive for alloantibodies and whether the CDC potential of these sera might be related to the amount of antibodies. **Figure 6** shows the CDC results for two patients in Cohorts A and B against the three target cells with sera whose complement activity was validated (**Supplementary Figure 9**). The whole kinetic study is shown in **Supplementary Figure 12**. In addition, corresponding mean fluorescence intensity (MFI) levels of anti-HLA antibodies against class I (B7, B44) and class II (DR103, DR8, DQB04, DQB05 and DPB02) molecules, present in those sera, are shown. For the two patients treated with PDC*lung01 alone (P6 and P7, **Figure 6A**) and the patient treated in combination with anti-PD-1 (P25, **Figure 6B**), whose sera were cytotoxic at V6, samples taken at earlier and later timepoints also showed cytotoxicity against both B-cell controls. It is interesting to note that one patient (P41) in Cohort B2, whose sera were negative at V3, V4, V5, and V6, became positive at V8 (**Figure 6B**). Furthermore, while the sera of patients P6 and P25 showed similar results at timepoints V6 and V8 against DR8+ (HD#01) and DR103+ (HD#02) B cells, the results of patient P7 sera showed a different profile. Indeed, P7 sera from five timepoints (V5, V6, V8, FU#1, and FU#2) showed antibody-mediated CDC against DR8+ B cells, while only three sera (V6, V8, and FU#1) were cytotoxic to DR103+ B cells. These results showed that despite similar MFI levels, the characteristics of anti-DR103 and anti-DR8 antibodies may differ significantly in terms of induction of CDC.

With regard to PDC*line cells, despite the use of patient samples containing high levels of antibodies targeting the highly expressed HLA molecules and the validation of complement functionality, alloantibodies remained unable to induce CDC towards these cells even when patients received the combination treatment. However, one sample from one timepoint (P7, FU#1) displayed a cytotoxicity activity just above the positivity threshold (22%).

Overall, sera from patients treated with anti-PD1 did not exhibit higher cytotoxicity against any of the cellular targets compared to sera from patients treated with PDC*lung01 alone.

### Inhibition of mCRPs does not increase CDC mediated by antibodies from patients treated with anti-PD-1

**Figure 7:**
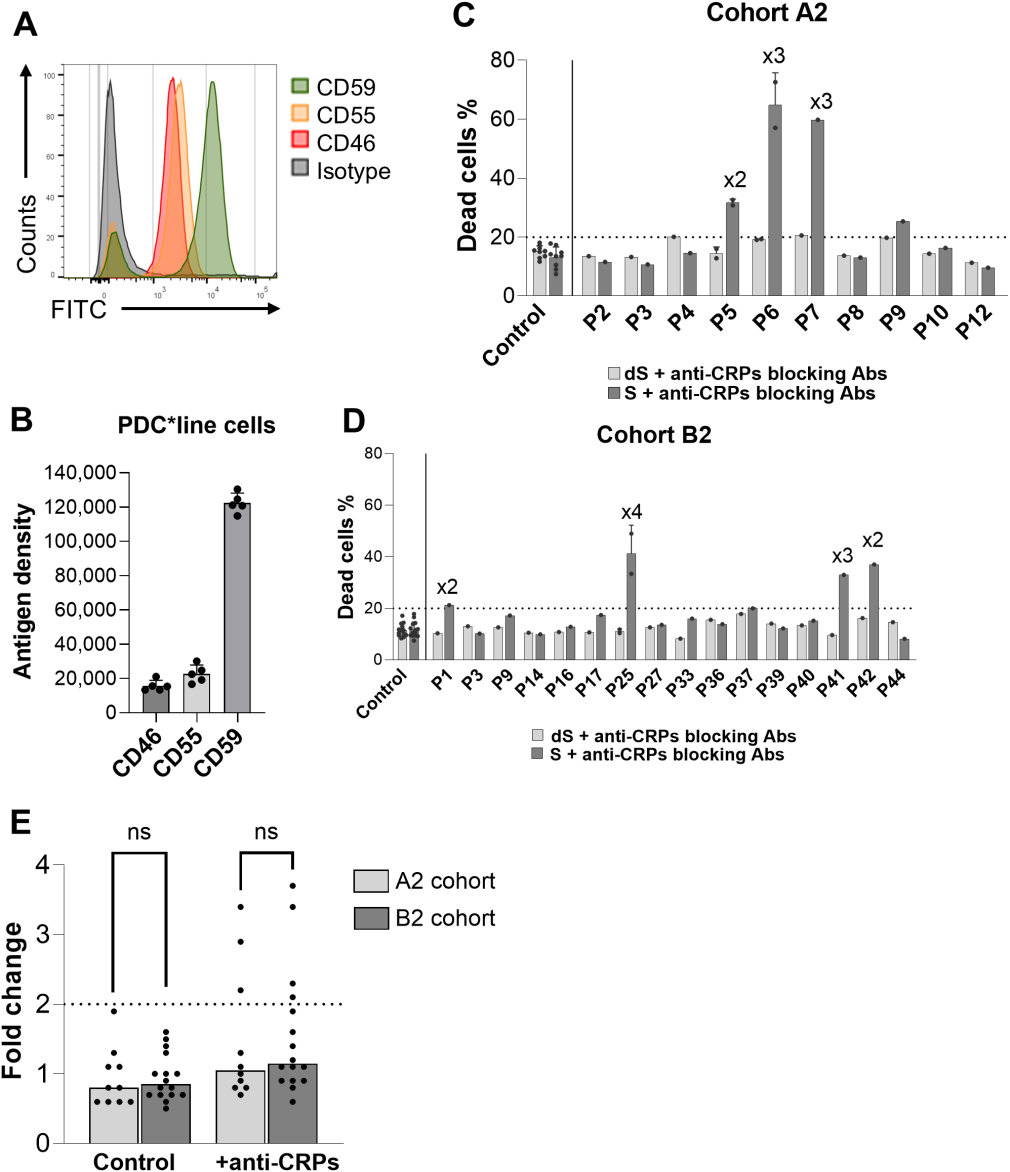
Effect of membrane complement regulatory proteins (mCRPs) blocking on CDC activity of patients’ sera. **(A)** Representative histograms of PDC*line cell labelling with anti-CD46 (red), -CD55 (orange), and -CD59 (green) antibodies. Cognate isotype was used as negative control (grey). **(B)** Quantification result expressed as CD46, CD55, and CD59 antigen density at the PDC*line cell surface (means +SD; n=5). **(C, D)** CDC results with cohort A2 **(C)** and B2 **(D)** patients (P) sera against PDC*line cells with anti-CD46, -CD55, and -CD59 blocking antibodies (anti-CRPs blocking Abs). dS: decomplemented serum, S: untreated serum. The dotted line is the 20% positivity threshold. The “xn” sign above the bar plots indicates the fold change in dead cells between the untreated serum and decomplemented serum conditions for the corresponding patient. Means +SD; n=1–2 for patients, n=9 to 14 for control sera depending of experiments. **(E)** Fold change of serum cytotoxicity between dS and S conditions in Cohort A2 (n=10) and B2 (n=16) with or without anti-CD46, -CD55, and -CD59 blocking antibodies. The horizontal dotted line indicates the twofold positivity threshold. The bars show the median values. t test was used to statistically compare results from A2 and B2 cohort for control and anti-CRPs conditions (ns, non-significant).

In order to better evaluate the CDC activity of patients’ sera from both cohorts against PDC*line cells, we used blocking antibodies against the three main membrane complement regulatory molecules (mCRP: CD46, CD55, and CD59) in our CDC assay. Indeed, as shown in **Figures 7A, B**, PDC*line cells express high levels of these molecules, particularly CD59, compared to B cells or monocytes (**Supplementary Figure 1B**). We observed that the addition of blocking antibodies against these mCRPs in the CDC assay with patients’ sera resulted in cell death of PDC*line cells with three and four patients’ sera treated with PDC*lung01 alone (Cohort A2) and in combination with anti-PD-1 (Cohort B2), respectively. All patients’ sera that were positive for CDC relative to B-cell controls without blocking mCRPs (**Figures 7, D**) were able to kill PDC*line cells when mCRPs were inhibited, confirming the cytotoxic properties of these samples. These results showed that, despite a more favorable context where PDC*line cells were sensitive to CDC, we did not observe enhancement of cytotoxic functionality of the alloantibodies generated following anti-PD-1 treatment (**Figure 7E**).

## Discussion

The remarkable clinical benefit observed in many patients treated with PD-1 receptor blocking antibodies is largely explained by the release of cytotoxic activity from pre-existing anti-tumor CD8+ T cells in the tumor bed, where its PD-L1/L2 ligands are well expressed by tumor cells or infiltrating myeloid cells (9).

However, little is known about the impact of PD-1 blockade on humoral immunity in general and on alloimmunization in particular, despite the widespread use of therapeutic antibodies for more than a decade (1) and more recently in the treatment of transplant patients developing cancer (3–5). Indeed, due to expression of PD-1 on Tfh cells and PD-L1/L2 on dendritic cells and B cells in the germinal center, it has been assumed that the transformation of B cells into antibody-secreting plasma cells could be positively influenced by anti-PD-1 treatment (33).

In this study, we have exploited a unique clinical situation where the triggering of humoral immune response against HLA molecules could be examined concomitantly with PD-1 blockade in humans (22). Indeed, to our knowledge, such analysis of the anti-HLA response in several patients after repeated injections of the same allogeneic dendritic cell-based vaccine in the presence or absence of therapeutic antibodies against PD-1 has never been described before. Importantly, by contrast to other studies describing the effect of anti-PD-1 in patients on humoral response, our control cohort (not receiving anti-PD-1) was composed of cancer patients instead of healthy controls in other studies (18, 20). It is interesting to note that patients included in Cohort B2 were at a more advanced stage (stage IV) without prior treatment, whereas those in Cohort A2 were at an earlier stage (stage II/III) but had undergone surgery and adjuvant therapy, with a withdrawal period of at least 4 weeks before receiving PDC*lung01 treatment. A minimum blood lymphocyte count of 1,000 cells/µl was required for all patients to ensure that their immune systems were not significantly compromised by disease or prior therapy. Immunological monitoring of leukocyte subpopulations, including B lymphocytes, during the clinical trial did not reveal any major abnormalities in either cohort (manuscript in preparation). In addition, analyses of allogeneic CD4^+^ lymphocyte responses (data not shown) and antigen-specific CD8^+^ responses (22) showed no differences between the cohorts. Therefore, the immune systems of the two patient cohorts can be considered comparable.

Our results showed that the generation of antibodies against HLA class I or II depended on the cumulative number of cells injected. In fact, apart from one patient, those treated with a low dose of PDC*lung01 corresponding to a cumulative total of 84 million cells did not develop an alloreactive immune response, even in the presence of pembrolizumab. In contrast, in the high dose cohort, anti-DRB1 antibodies began to be detected in a few patients after the first or second administration of the drug product (corresponding to 140 or 240 million cells). In a previous clinical trial in melanoma (26) (NCT01863108), where up to 180 million cells were injected into three patients, we did not observe alloimmune response. In the melanoma study, cells were injected only subcutaneously, while in the PDC-LUNG-01 trial the intravenous route was added, likely increasing cellular immunogenicity.

It should be noted that the percentage of patients who developed an alloimmune response differed greatly between the A2 and B2 Cohorts. Indeed, while 50% (6/12) and 91.7% (11/12) of patients treated with PDC*lung01 alone were positive for anti-class I and anti-class II antibodies respectively, only 29.8% (14/47) and 51.1% (24/47) of patients were positive when receiving the drug combination (Chi2 tests: ns for anti-class I, p=0.0106 for anti-HLA-class II). We decided to further compare the HLA-DRB1 allele distribution of patients in Cohorts A2 and B2 (**Supplementary Figure 4**). This distribution was indeed different between cohorts, strongly suggesting that the difference in immunization could be due to the heterogeneity of incompatibility between patients and PDC*line cells. However, this hypothesis would require further investigations to be confirmed.

Overall, the characteristics of the humoral immune response (type of targeted HLA molecules, initiation of antibody secretion, intensity of anti-HLA levels, maintenance and duration of alloantibodies in serum) were similar between cohorts, indicating an absence of positive effect of PD-1 blockade, contrary to what was expected. This conclusion should, however, be interpreted with caution due to the unequal number of patients analyzed; 12 in the monotherapy group versus 48 in the combination with anti–PD-1 group. Several studies in different human contexts have reported increased activation and proliferation of Tfh or Tfr (34) (a subpopulation of regulatory CD4+ T cells regulating antibody responses) associated with changes in B cells in germinal centers or circulating plasmablasts and upregulation of the humoral immune response at some timepoints (16, 18). These observations have also been corroborated by experimental models in which PD-1 has been shown to suppress Tfh cell differentiation (13) and, consequently, PD-1 blockade has led to Tfh expansion and increased humoral responses (14–17). However, in other reports, in cancer patients undergoing anti-PD-1 treatment, little or no change was observed in the level and characteristics of antiviral antibodies after a viral vaccination against SARS-Cov-2 (21) or influenza virus (20). It is important to note here that most of the studies analyzed the antiviral response in the context of a booster vaccination. In our study, patients had never received PDC*lung01 treatment or other allogeneic cell therapies, which highlights the originality of the results. Thus, we observed a *de novo* humoral response in patients who had never received anti-PD-1 treatment before.

We also sought to determine whether anti-HLA antibody production could be correlated with antigen-specific CD8^+^ T cell responses or clinical activity. No allogeneic lymphocyte response to PDC*line cells was observed in patients who developed alloimmunization (**Supplementary Figure 13**), regardless of cohort. An antigen-specific CD8^+^ T-cell response was detected in a large proportion of patients in all cohorts (50% to 67%) (22) and was not correlated with anti-HLA antibody production. In B2 Cohort where clinical activity can be analysed, the confirmed objective response rate reached 51%, with a 9-month progression-free survival (PFS) rate of 47%, showing a correlation between antitumor response and PFS duration. Again, no relationship was observed between the clinical activity of PDC*lung01 combined with anti-PD-1 therapy and the occurrence of alloimmunization.

We also evaluated whether PD-1 blockade could affect the functionality of these antibodies. Anti-HLA antibodies are known to cause antibody-mediated organ rejection after transplantation via the complement cascade and are responsible for many episodes of acute rejection in kidney transplant patients (35). We therefore explored the antibody-mediated CDC functionality of anti-HLA antibodies from the serum of patients from the two cohorts.

We observed that despite high levels of anti-HLA antibodies and positive trends in cytotoxic potential observed with assays used in HLA laboratories, few patients actually had antibodies that caused cell death of positive control cells in a cytotoxicity assay using human complement. There were not more patient sera with cytotoxic activity in the cohort with anti-PD-1 treatment. Furthermore, after restoring the sensitivity of PDC*line cells in the presence of mCRP inhibitors, we did not observe that anti-PD1 blockade enhanced the cytotoxic potential of anti-HLA antibodies, even though the context was more favorable than with B-cell controls. Indeed, with PDC*line cells, all alloantibodies can bind to all HLA molecules that are largely expressed on the membrane surface and as a result can exert fully their cytotoxic function. Recently, it has been shown that antibodies generated in patients treated with anti-PD-1 have reduced sialylation, affecting the affinity of the molecules (18). However, previously, in a mouse model, no maturation of antibody affinity was observed after a primary/booster vaccination regimen (16). Further examination of the nature of the isotype, glycosylation, and sialylation of patients’ anti-HLA antibodies could help detect potential differences at protein level in the humoral immune response generated in the presence or absence of anti-PD-1 treatment. Regarding cellular immune response, we did not see any difference of allogeneic cell proliferation of patients’ PBMCs between both cohorts (**Supplementary Figure 13**), whereas the magnitude of antitumor specific immune response after vaccination appeared linked to the PD-1 blockade (22).

Altogether these results showed that, in the specific situation of inducing an allogeneic humoral response following allogeneic dendritic cell-based cancer vaccine treatment, PD-1 blockade surprisingly does not improve either the generation of alloantibodies or their functionality, which should allow for a better understanding of the mechanisms involved in graft failure or rejection in solid transplant recipients subjected to PD-1 blockade. This unexpected outcome reveals an apparent paradox: PD-1 blockade enhances CD8^+^ antitumor responses while sparing allogeneic humoral responses, highlighting the immune system’s ability to produce divergent, antigen-specific responses even under systemic immunomodulation. Recent studies illustrate this principle: the intestinal immune system triggers pro-inflammatory or tolerogenic responses to commensal bacteria based on distinct motility signatures and flagellin disposition (36). Similarly, our data suggest that PD-1 blockade amplifies tumor-specific cytotoxic responses (22) without disrupting alloimmunization in the context of allogeneic dendritic cell-based vaccines. This decoupling reflects precise discrimination of molecular signatures and cellular interactions rather than uniform disinhibition of checkpoints.

## Data Availability

The dataset presented in this article are available upon reasonable request for research purposes aligned with the clinical trial protocol. Interested parties should direct their requests to Dr Joël Plumas. Requests to access the datasets should be directed to joel.plumas@outlook.fr.

## Supplementary material

Supplementary Figure 1 HLA molecules and membrane complement regulatory protein (mCRP) quantification on primary B cells and monocytes.

Supplementary Figure 2 HLA mismatch overview between PDC*line and patient HLA typing.

Supplementary Figure 3 Anti-HLA humoral response of Patient P5 of cohort A1.

Supplementary Figure 4 DRB1 allele distribution in A2 and B2 cohorts

Supplementary Figure 5 Anti-HLA humoral response against HLA-DQB1 and DPB1 molecules.

Supplementary Figure 6 Comparison of mean MFI levels of anti-HLA humoral response in patients from cohorts A2 and B2 over time.

Supplementary Figure 7 Representative cytofluorimetry results after Complement-dependent cytotoxicity (CDC) experiments.

Supplementary Figure 8 Complement-dependent cytotoxicity (CDC) assay on irradiated and non-irradiated PDC*line cells.

Supplementary Figure 9 Patients’ complement validation.

Supplementary Figure 10 Recognition of PDC*line cells and control B cells by anti-HLA antibodies of Cohort A2 and B2 patients.

Supplementary Figure 11 Complement-dependent cytotoxicity (CDC) activity of cohort A2 **(A)** and B2 **(B)** patients’ sera against unmatched B cells (HD#03).

Supplementary Figure 12 Kinetics of CDC activity according to anti-HLA levels.

Supplementary Figure 13 Cellular alloreactivity of patients with humoral responses in A2 **(A)** and B2 **(B)** cohorts.

Supplementary Table 1 Patient Demographics.

Supplementary Table 2 HLA Typing of healthy donors (HD).

Supplementary Table 3 C3d deposition and Lymphocytoxicity (LCT) results.

## References

[1] Beaver JA Hazarika M Mulkey F Mushti S Chen H He K . Patients with melanoma treated with an anti-PD-1 antibody beyond RECIST progression: a US Food and Drug Administration pooled analysis. Lancet Oncol. (2018) 19:229−39. doi: 10.1016/S1470-2045(17)30846-X, PMID: 29361469 PMC5806609

[2] Mazzoli G Nichetti F Shitara K Cohen R Lonardi S Cremolini C . Comparative efficacy of PD-1 blockade in patients with dMMR/MSI-H metastatic colorectal or gastric cancer: a global retrospective study. ESMO Gastrointest Oncol 1 mars. (2024) 3:100037. doi: 10.1016/j.esmogo.2023.100037, PMID: 41648740 PMC12836713

[3] Aguirre LE Guzman ME Lopes G Hurley J . Immune checkpoint inhibitors and the risk of allograft rejection: A comprehensive analysis on an emerging issue. Oncologist. (2019) 24:394−401. doi: 10.1634/theoncologist.2018-0195, PMID: 30413665 PMC6519766

[4] Portuguese AJ Tykodi SS Blosser CD Gooley TA Thompson JA Hall ET . Immune checkpoint inhibitor use in solid organ transplant recipients: A systematic review. J Natl Compr Canc Netw. (2022) 20:406–416.e11. doi: 10.6004/jnccn.2022.7009, PMID: 35390767

[5] D’Izarny-Gargas T Durrbach A Zaidan M . Efficacy and tolerance of immune checkpoint inhibitors in transplant patients with cancer: A systematic review. Am J Transplant. (2020) 20:2457−65. doi: 10.1111/ajt.15811, PMID: 32027461

[6] Van Meerhaeghe T Murakami N Le Moine A Brouard S Sprangers B Degauque N . Fine-tuning tumor- and allo-immunity: advances in the use of immune checkpoint inhibitors in kidney transplant recipients. Clin Kidney J. (2024) 17:sfae061. doi: 10.1093/ckj/sfae061, PMID: 38606169 PMC11008728

[7] Barbir EB Abdulmoneim S Dudek AZ Kukla A . Immune checkpoint inhibitor therapy for kidney transplant recipients - A review of potential complications and management strategies. Transpl Int. (2024) 37:13322. doi: 10.3389/ti.2024.13322, PMID: 39479217 PMC11521864

[8] Keir ME Butte MJ Freeman GJ Sharpe AH . PD-1 and its ligands in tolerance and immunity. Annu Rev Immunol. (2008) 26:677−704. doi: 10.1146/annurev.immunol.26.021607.090331, PMID: 18173375 PMC10637733

[9] Zou W Chen L . Inhibitory B7-family molecules in the tumour microenvironment. Nat Rev Immunol. (2008) 8:467−77. doi: 10.1038/nri2326, PMID: 18500231

[10] Schaerli P Willimann K Lang AB Lipp M Loetscher P Moser B . Cxc chemokine receptor 5 expression defines follicular homing T cells with B cell helper function. J Exp Med. (2000) 192:1553−62. doi: 10.1084/jem.192.11.1553, PMID: 11104798 PMC2193097

[11] Breitfeld D Ohl L Kremmer E Ellwart J Sallusto F Lipp M . Follicular B helper T cells express cxc chemokine receptor 5, localize to B cell follicles, and support immunoglobulin production. J Exp Med. (2000) 192:1545−52. doi: 10.1084/jem.192.11.1545, PMID: 11104797 PMC2193094

[12] Haynes NM Allen CDC Lesley R Ansel KM Killeen N Cyster JG . Role of CXCR5 and CCR7 in follicular th cell positioning and appearance of a programmed cell death gene-1High germinal center-associated subpopulation1. J Immunol. (2007) 179:5099−108. doi: 10.4049/jimmunol.179.8.5099, PMID: 17911595

[13] Sage PT Schildberg FA Sobel RA Kuchroo VK Freeman GJ Sharpe AH . Dendritic cell PD-L1 limits autoimmunity and follicular T cell differentiation and function. J Immunol. (2018) 200:2592−602. doi: 10.4049/jimmunol.1701231, PMID: 29531164 PMC6054131

[14] Hams E McCarron MJ Amu S Yagita H Azuma M Chen L . Blockade of B7-H1 (Programmed death ligand 1) enhances humoral immunity by positively regulating the generation of T follicular helper cells. J Immunol. (2011) 186:5648−55. doi: 10.4049/jimmunol.1003161, PMID: 21490158

[15] Sánchez-Alonso S Setti-Jerez G Arroyo M Hernández T Martos MI Sánchez-Torres JM . A new role for circulating T follicular helper cells in humoral response to anti-PD-1 therapy. J Immunother Cancer. (2020) 8:e001187. doi: 10.1136/jitc-2020-001187, PMID: 32900863 PMC7478024

[16] Zhang M Xia L Yang Y Liu S Ji P Wang S . PD-1 blockade augments humoral immunity through ICOS-mediated CD4+ T cell instruction. Int Immunopharmacol. (2019) 66:127−38. doi: 10.1016/j.intimp.2018.10.045, PMID: 30448635

[17] Velu V Titanji K Zhu B Husain S Pladevega A Lai L . Enhancing SIV-specific immunity *in vivo* by PD-1 blockade. Nature. (2009) 458:206−10. doi: 10.1038/nature07662, PMID: 19078956 PMC2753387

[18] Herati RS Knorr DA Vella LA Silva LV Chilukuri L Apostolidis SA . PD-1 directed immunotherapy alters Tfh and humoral immune responses to seasonal influenza vaccine. Nat Immunol. (2022) 23:1183−92. doi: 10.1038/s41590-022-01274-3, PMID: 35902637 PMC9880663

[19] Barth DA Stanzer S Spiegelberg JA Bauernhofer T Absenger G Szkandera J . Patterns of peripheral blood B-cell subtypes are associated with treatment response in patients treated with immune checkpoint inhibitors: A prospective longitudinal pan-cancer study. Front Immunol. (2022) 13. Available online at: https://www.frontiersin.org/journals/immunology/articles/10.3389/fimmu.2022.840207/full (Accessed December 01, 2025). 10.3389/fimmu.2022.840207 PMC9010871 35432362

[20] Läubli H Balmelli C Kaufmann L Stanczak M Syedbasha M Vogt D . Influenza vaccination of cancer patients during PD-1 blockade induces serological protection but may raise the risk for immune-related adverse events. J Immunother Cancer. (2018) 6:40. doi: 10.1186/s40425-018-0353-7, PMID: 29789020 PMC5964701

[21] Valanparambil RM Carlisle J Linderman SL Akthar A Millett RL Lai L . Antibody response to COVID-19 mRNA vaccine in patients with lung cancer after primary immunization and booster: reactivity to the SARS-coV-2 WT virus and omicron variant. J Clin Oncol. (2022) 40:3808−16. doi: 10.1200/JCO.21.02986, PMID: 35759727 PMC9671759

[22] Vansteenkiste J Demedts I Cuppens K Pons-Tostivint E Wauters E Borm FJ . Innovative therapeutic cancer vaccine PDC∗lung01 with or without anti-PD-1: an open-label, dose-escalation phase I/II study in non-small-cell lung cancer. ESMO Open. (2025) 10:105844. doi: 10.1016/j.esmoop.2025.105844, PMID: 41101155 PMC12550581

[23] Aspord C Leccia MT Salameire D Laurin D Chaperot L Charles J . HLA-A*0201 + Plasmacytoid dendritic cells provide a cell-based immunotherapy for melanoma patients. J Invest Dermatol. (2012) 132:2395−406. doi: 10.1038/jid.2012.152, PMID: 22696054

[24] Hannani D Leplus E Laurin D Caulier B Aspord C Madelon N . A new plasmacytoid dendritic cell-based vaccine in combination with anti-PD-1 expands the tumor-specific CD8+ T cells of lung cancer patients. Int J Mol Sci. (2023) 24:1897. doi: 10.3390/ijms24031897, PMID: 36768214 PMC9915756

[25] Plumas J . Harnessing dendritic cells for innovative therapeutic cancer vaccines. Curr Opin Oncol. (2022) 34:161−8. doi: 10.1097/CCO.0000000000000815, PMID: 34930882

[26] Charles J Chaperot L Hannani D Costa JB Templier I Trabelsi S . An innovative plasmacytoid dendritic cell line-based cancer vaccine primes and expands antitumor T-cells in melanoma patients in a first-in-human trial. OncoImmunology. (2020) 9:1738812. doi: 10.1080/2162402X.2020.1738812, PMID: 32313721 PMC7153838

[27] Bertrand D Farce F Laurent C Hamelin F François A Guerrot D . Comparison of two luminex single-antigen bead flow cytometry assays for detection of donor-specific antibodies after renal transplantation. Transplantation. (2019) 103:597−603. doi: 10.1097/TP.0000000000002351, PMID: 29965954

[28] Jacob MC Agrawal S Chaperot L Giroux C Gressin R Le Marc’Hadour F . Quantification of cellular adhesion molecules on Malignant B cells from non-Hodgkin’s lymphoma. Leukemia. (1999) 13:1428−33. doi: 10.1038/sj.leu.2401517, PMID: 10482995

[29] Smith KB Ellis SA . Standardisation of a procedure for quantifying surface antigens by indirect immunofluorescence. J Immunol Methods. (1999) 228:29−36. doi: 10.1016/S0022-1759(99)00087-3, PMID: 10556540

[30] Lecoeur H Ledru E Prévost MC Gougeon ML . Strategies for phenotyping apoptotic peripheral human lymphocytes comparing ISNT, annexin-V and 7-AAD cytofluorometric staining methods. J Immunol Methods. (1997) 209:111−23. doi: 10.1016/S0022-1759(97)00138-5, PMID: 9461328

[31] Manches O Lui G Chaperot L Gressin R Molens JP Jacob MC . *In vitro* mechanisms of action of rituximab on primary non-Hodgkin lymphomas. Blood. (2003) 101:949−54. doi: 10.1182/blood-2002-02-0469, PMID: 12393572

[32] Navas A Molina J Agüera ML Guler I Jurado A Rodríguez-Benot A . Characterization of the C1q-binding ability and the igG1–4 subclass profile of preformed anti-HLA antibodies by solid-phase assays. Front Immunol. (2019) 10. Available online at: https://www.frontiersin.org/journals/immunology/articles/10.3389/fimmu.2019.01712/full (Accessed December 01, 2025). 10.3389/fimmu.2019.01712 PMC6687874 31428086

[33] Crotty S . T follicular helper cell biology: A decade of discovery and diseases. Immunity. (2019) 50:1132−48. doi: 10.1016/j.immuni.2019.04.011, PMID: 31117010 PMC6532429

[34] Sage PT Sharpe AH . T follicular regulatory cells. Immunol Rev. (2016) 271:246−59. doi: 10.1111/imr.12411, PMID: 27088919

[35] Loupy A Lefaucheur C Vernerey D Prugger C Huyen JPDV Mooney N . Complement-binding anti-HLA antibodies and kidney-allograft survival. New Engl J Med. (2013) 369:1215−26. doi: 10.1056/NEJMoa1302506, PMID: 24066742

[36] Duck LW Jennings MS Hsu JS Adeboboye CF Morgan EL Coger KJ . Divergent immune responses to commensal bacteria bearing distinct motility signatures. Sci Immunol. (2025) 10:eadp8843. doi: 10.1126/sciimmunol.adp8843, PMID: 41417926 PMC12875389

