# Supplementary material for: PD-1 blockade does not enhance alloimmunization after allogeneic dendritic cell vaccination in cancer patients
Source: Front Immunol. 2026 Mar 4;17:1763434. doi: 10.3389/fimmu.2026.1763434 (PMC12995621; doi:10.3389/fimmu.2026.1763434)
Supplement: Supplementary Figure 1 — HLA molecules and membrane complement regulatory protein (mCRP) quantification on primary B cells and monocytes. [file DataSheet1.pdf]

PD-1 blockade does not enhance alloimmunization after allogeneic dendritic cell vaccination in cancer patients

Planel S. et al.

Supplementary materials

Materials and Methods

Mixed leukocyte Reaction

When patients generated anti-HLA antibodies, the cellular alloreactivity was measured using available blood samples collected at screening, V7, V8, and V9 visits: one patient of Cohort A1, 11 patients of Cohort A2, and 18 patients from Cohort B2. One patient of Cohort B2 who was positive for anti-HLA was not assessed.

The allogeneic cell proliferation of patients' PBMCs was evaluated with a Mixed Leucocyte Reaction based on EdU incorporation in replicating DNA after a 6-day coculture (C10499, Invitrogen) following the recommendations of the manufacturer. The coculture of non-irradiated PBMCs with the same irradiated autologous PBMCs was used as a negative control to assess the basal cell proliferation.

C3d binding assay

The C3d-binding assay was performed using the Luminex technology coupled to LIFECODES® C3d Detection and LIFECODES LSA™ Class I and II kits from Werfen according to the manufacturer's instructions. Results were expressed as mean fluorescence intensity (MFI) and positivity was determined using MatchIt! Antibody Software (Immucor Inc., Stamford, USA).

Lymphocytotoxicity assay

T cells for lymphocytotoxicity assay were isolated from PBMCs with EasySep™ Human CD3 Positive selection Kit II from StemCell. B cells were purified from PBMCs using EasySep™ Human B Cell Enrichment Kit from StemCell. Two thousand isolated T or B cells from HD#04, HD#05, HD#06, or HD#07 in 1 µL of RPMI were distributed in each well of Terasaki plates (One Lambda, Inc., Canoga Park, California, USA) followed by 1 µL of patient serum. After incubation at 21°C for 45 minutes, 5 µL of rabbit complement (Cedarlane) were added, followed by incubation for another 90 minutes. Then, 2 µL of a stain-quench agent (FluoroQuench™, One Lambda, Inc.) was added. The reaction was visualized under inverted fluorescence microscopy (Leitz Wetzlar, Zeiss) and a 10× objective lens. The positivity was evaluated by measuring the percentage of lysed cells (colored in red) versus all living cells (colored in green). For result validation, anti-B cells (One lambda), anti-T cells (One lambda) controls, IgM control (anti-ß2-microglobuline, BD Biosciences), negative and positive homemade control antibodies were used. Results were described using scores from 0 to 8 based on the American Society for histocompatibility and immunogenetics reading standards. The reaction was considered positive when the score was equal to or greater than 2. To determine the contribution of IgM and IgG subtypes to cytotoxicity, a volume of the tested patient's serum was pretreated in parallel with a Dithiothreitol solution (DTT, Sigma) at a final concentration of 50 mM for 20 minutes at 37°C. After treatment, the serum was placed in Terasaki plate for an LCT assay as described above with untreated sera.

Supplementary Figures

__Figure S1__

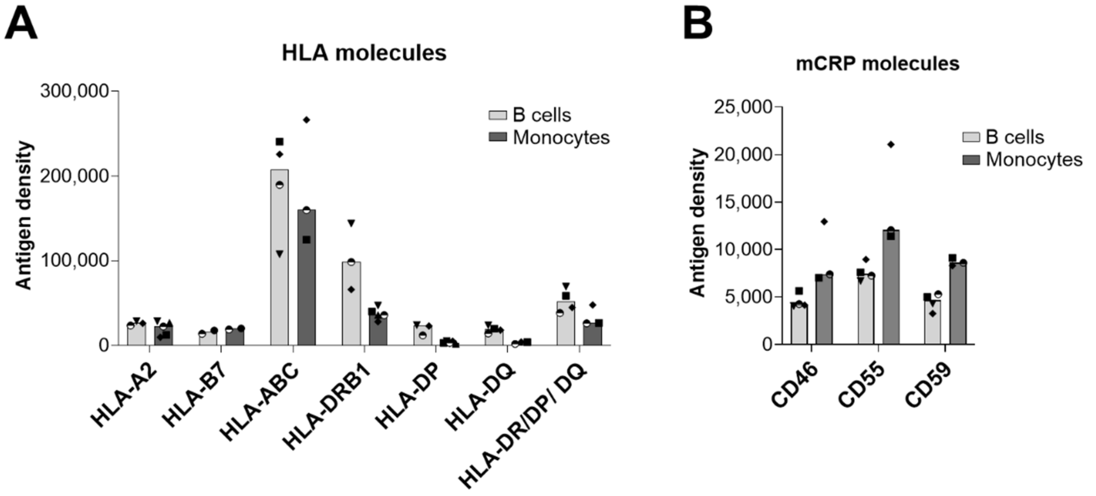

Figure S1. HLA molecules and membrane complement regulatory protein (mCRP) quantification on primary B cells and monocytes. (**A**) Quantification of HLA-A2, -B7, -ABC, -DRB1, -DP, -DQ, -DR/DP/DQ on primary B cells and monocytes using QIFIKIT assay. The medians are shown (n=2-4 for B cells and n=2-5 for monocytes). (**B**) Quantification of CD46, CD55 and CD59 on primary B cells and monocytes using QIFIKIT assay. The medians are shown (n=4 for B cells, n=3 for monocytes).

Figure S2

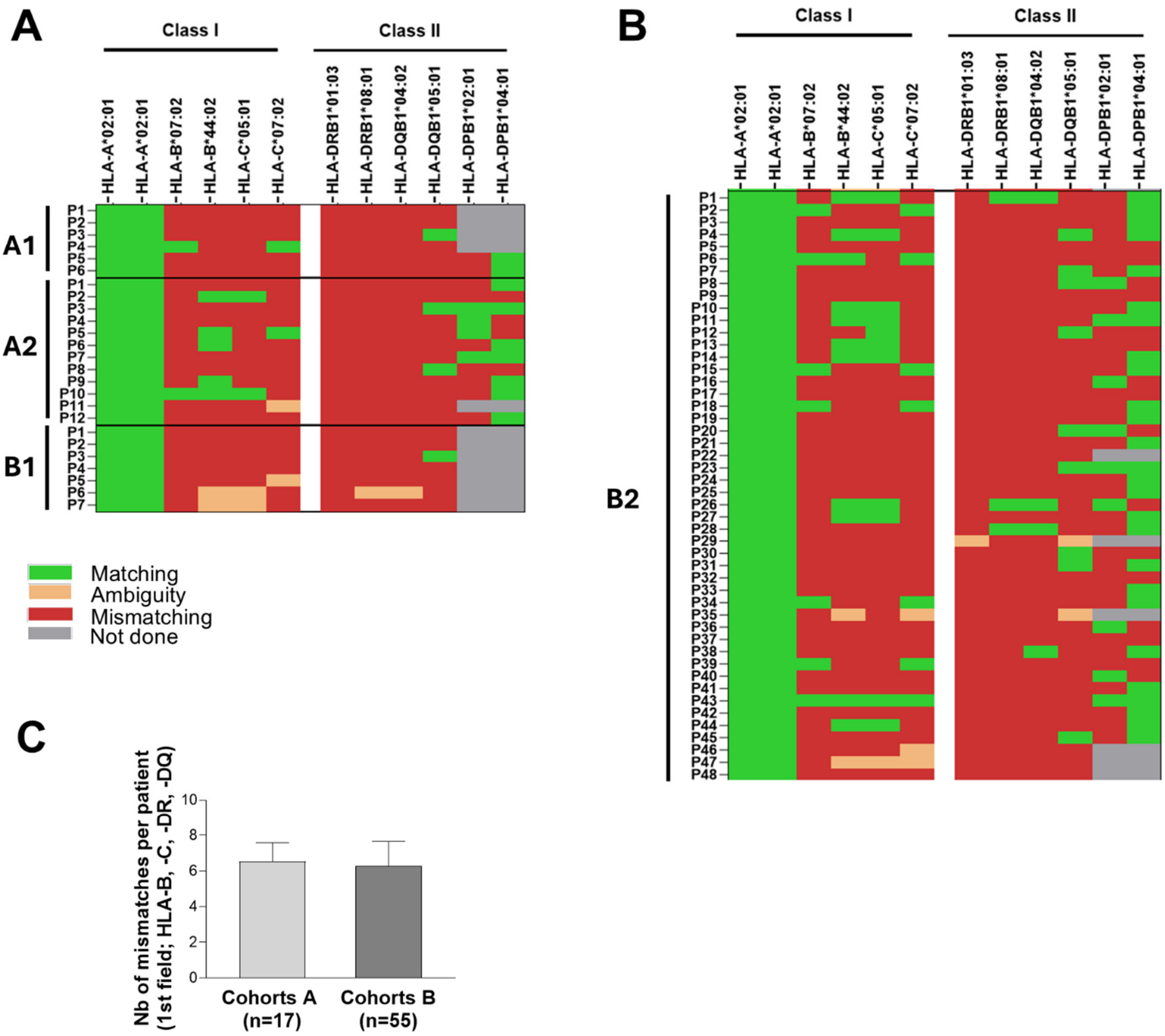

Figure S2. HLA mismatch overview between PDC*line and patient HLA typing. (**A**) HLA mismatch overview between PDC*line cells and patients of A1, A2 and B1 cohorts. Five out of 6 patients of cohort A1 and the 7 patients of cohort B2 had only 1st field (2 digits) sequencing resolution, whereas the other patients had 2nd field (4 digits) sequencing resolution. (**B**) HLA mismatch overview between PDC*line cells and cohort B2 patients. For A and B, « Matching » (Green) indicates that the patient's HLA typing is similar to that of PDC*line cells. « Ambiguity » (orange) indicates the impossibility to conclude. « Mismatching » (red) indicates that the patient's HLA typing and that of PDC*line cells are different. « Not done » (Grey) indicates that the patient's HLA typing is not available. (**C**) Number of mismatches per patients at 1st field (2 digits) in cohort A (cohort A1+A2) and B (cohort B1 + B2). The means +SD are shown.

Figure S3

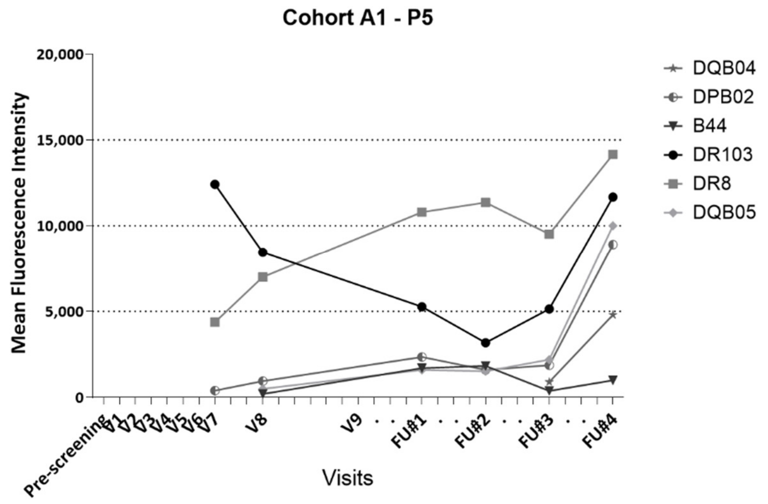

Figure S3. Anti-HLA humoral response of Patient P5 of cohort A1. The detection of anti-HLA Class I and anti-HLA Class II antibodies was evaluated overtime using Lifecodes Single antigen assay and Luminex technology. V=visit; FU=Follow-Up. The last injection of PDC*lung01 was at V6.

Figure S4

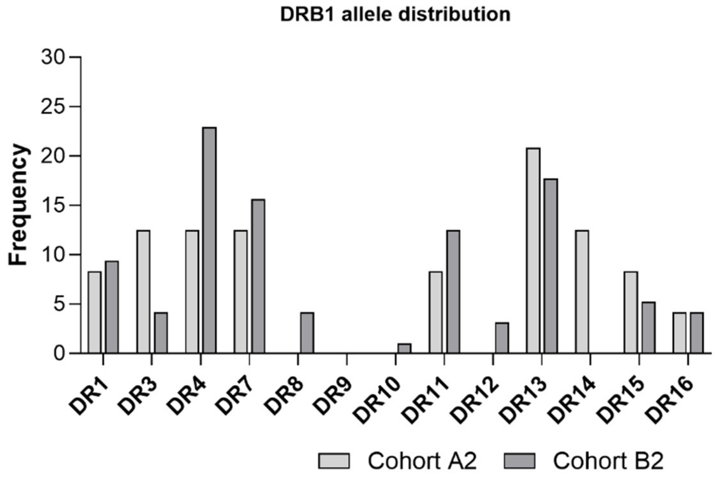

Figure S4. DRB1 allele distribution in A2 and B2 cohorts. See supplementary Figure 3 for methods. The frequencies of each DRB1 allele were calculated within patients of A2 and B2 cohorts.

Figure S5

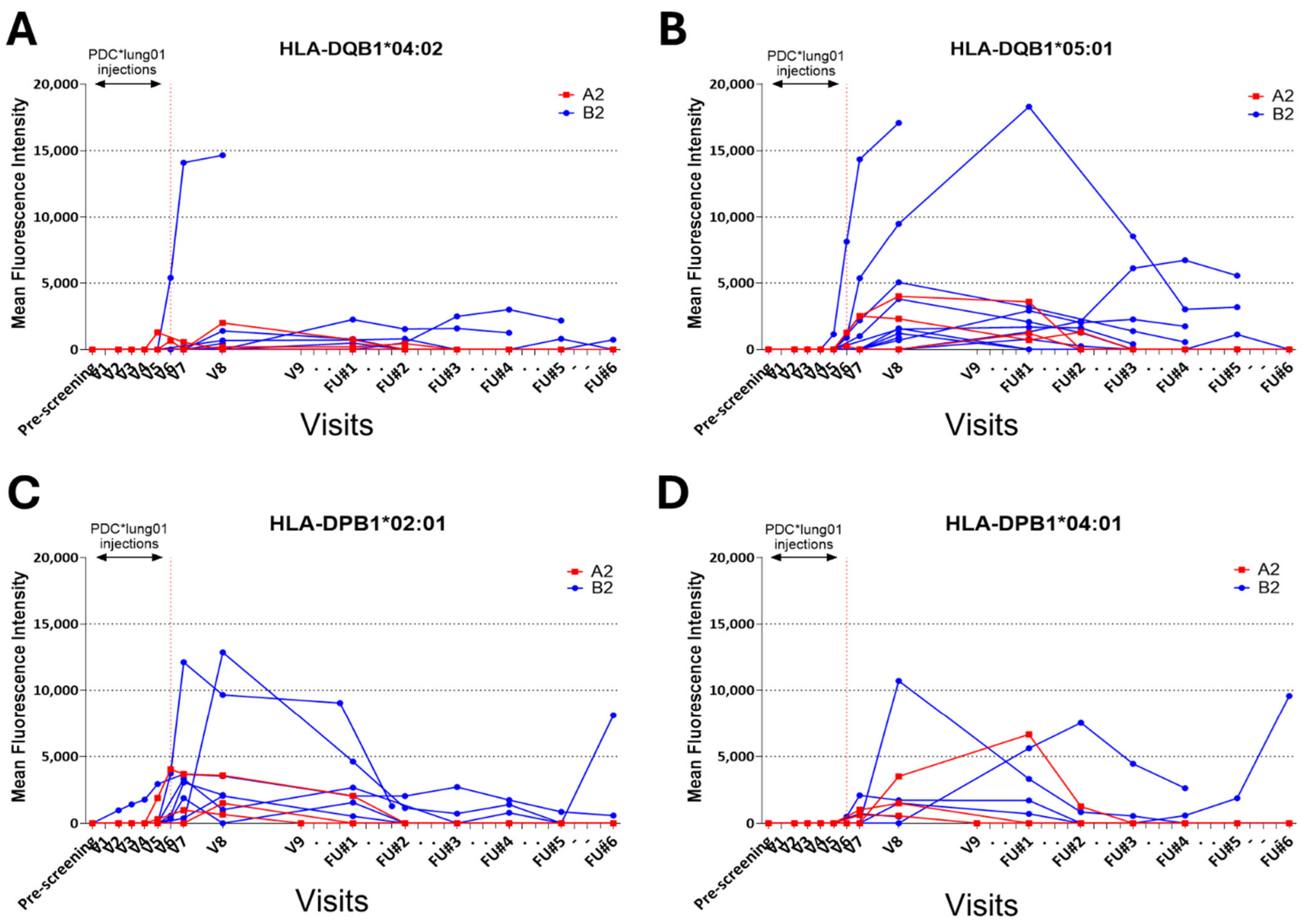

Figure S5. Anti-HLA humoral response against HLA-DQB1 and DPB1 molecules. HLA-DQB1 *04:02 (**A**), HLA-DQB1*05:01 (**B**), HLA-DPB1*02:01 (**C**), HLA-DPB1*04:01 (**D**) of cohort A2 (n=12) and B2 patients (n=48). The detection of anti-HLA Class II antibodies was performed over time in the sera of patients of cohort A2 (red) and B2 (blue) using Lifecodes Single antigen assay and Luminex technology. V=visit; FU=Follow-Up.

Figure S6

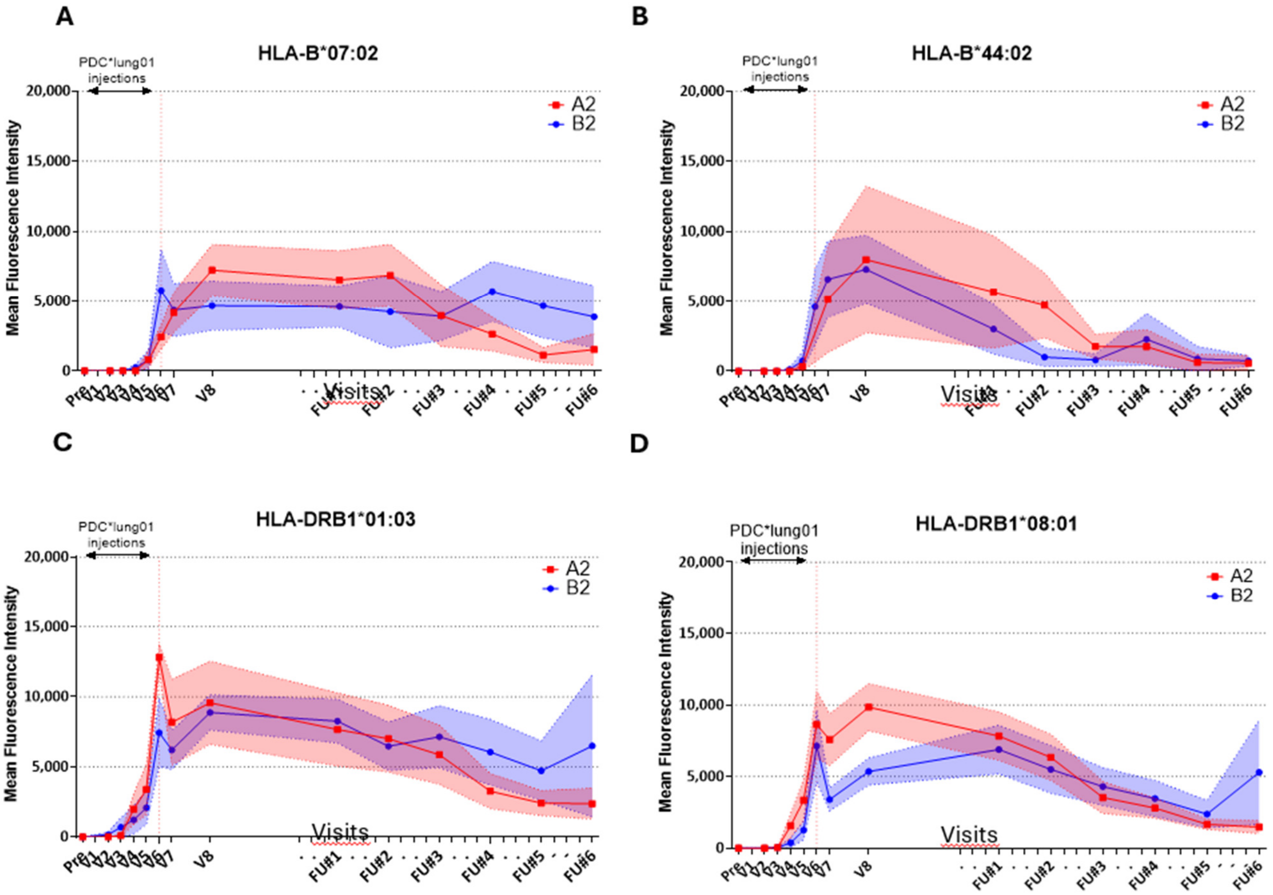

Figure S6. Comparison of mean MFI levels of anti-HLA humoral response in patients from cohorts A2 and B2 over time. The detection of anti-HLA Class I (**A, B**) and -HLA Class II (**C, D**) antibodies was performed at each timepoint in the sera of patients of cohort A2 (red) and B2 (blue). Only the most represented HLA molecules are shown: HLA-B*07:02 (B7; A), HLA-B*44:02 (B44; B), HLA-DRB1*01:03 (DR103; C), HLA-DRB1*08:01 (DR8; D). The lines represent the means of MFI for each HLA molecules at each visit (see individual measurements in Figure 2), and the colored area is the SEM. V=visit; FU=Follow-Up.

Figure S7

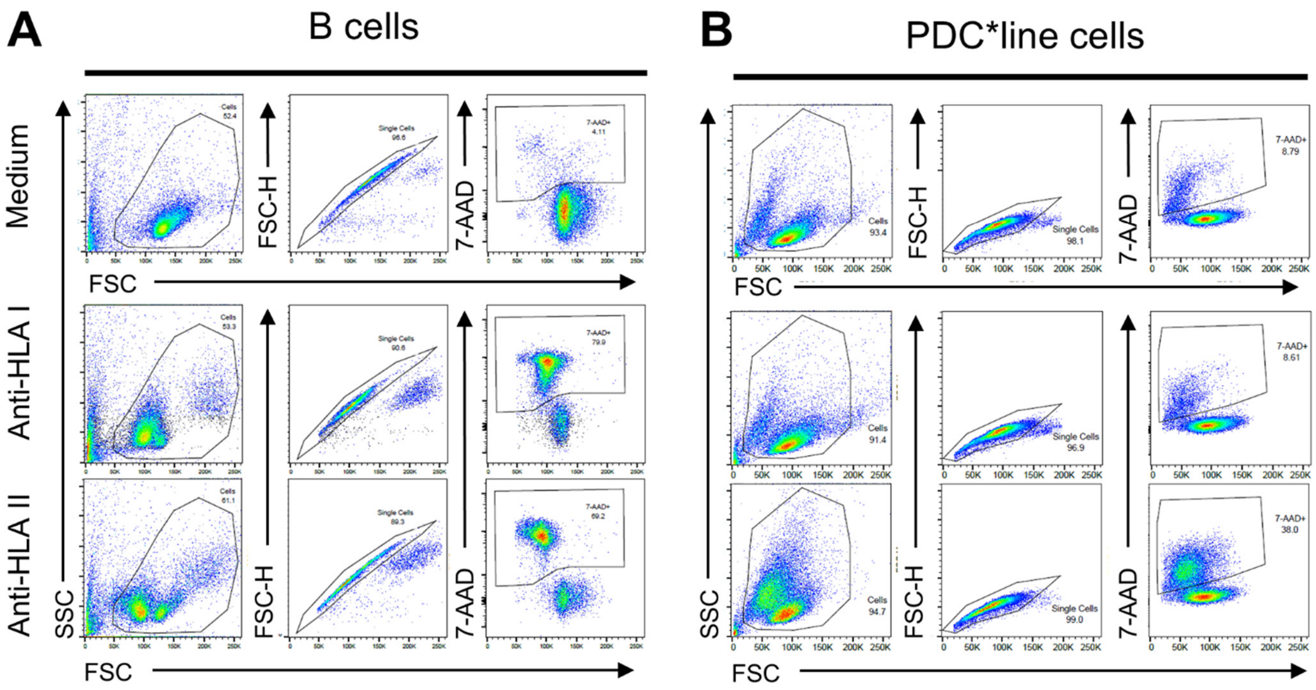

Figure S7. Representative cytofluorimetry results after CDC experiments. Primary B cells (**A**) and PDC*line cells (**B**) were subjected to CDC experiments with control anti-HLA Class I or Class II antibodies in the presence of human serum as source of complement for 1h at 37°C. Dead cells were identified by 7-AAD labelling. Fluorescence was acquired with flow cytometer and analyses performed with FlowJo software. The gating strategy consisted in selecting cells based on their morphology (SSC versus FSC, left panels), selecting single cells (FSC-H versus FSC-A, middle panels), and analyzing the proportion of 7-AAD-positive dead cells (right panels).

Figure S8

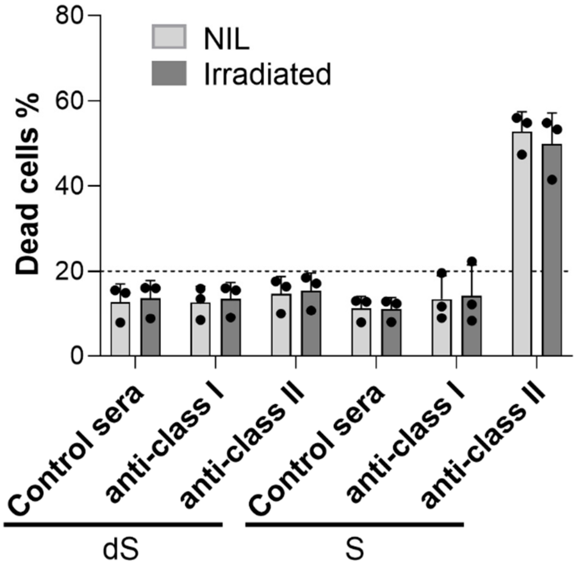

Figure S8. Complement-dependent cytotoxicity (CDC) assay on irradiated and non-irradiated PDC*line cells. CDC assay was performed with decomplemented serum (dS) or untreated serum (S) on non-irradiated (NIL) and 60 Gy-irradiated PDC*line cells. The dotted line is the 20% positivity threshold. The means +SD are presented (n=3).

Figure S9

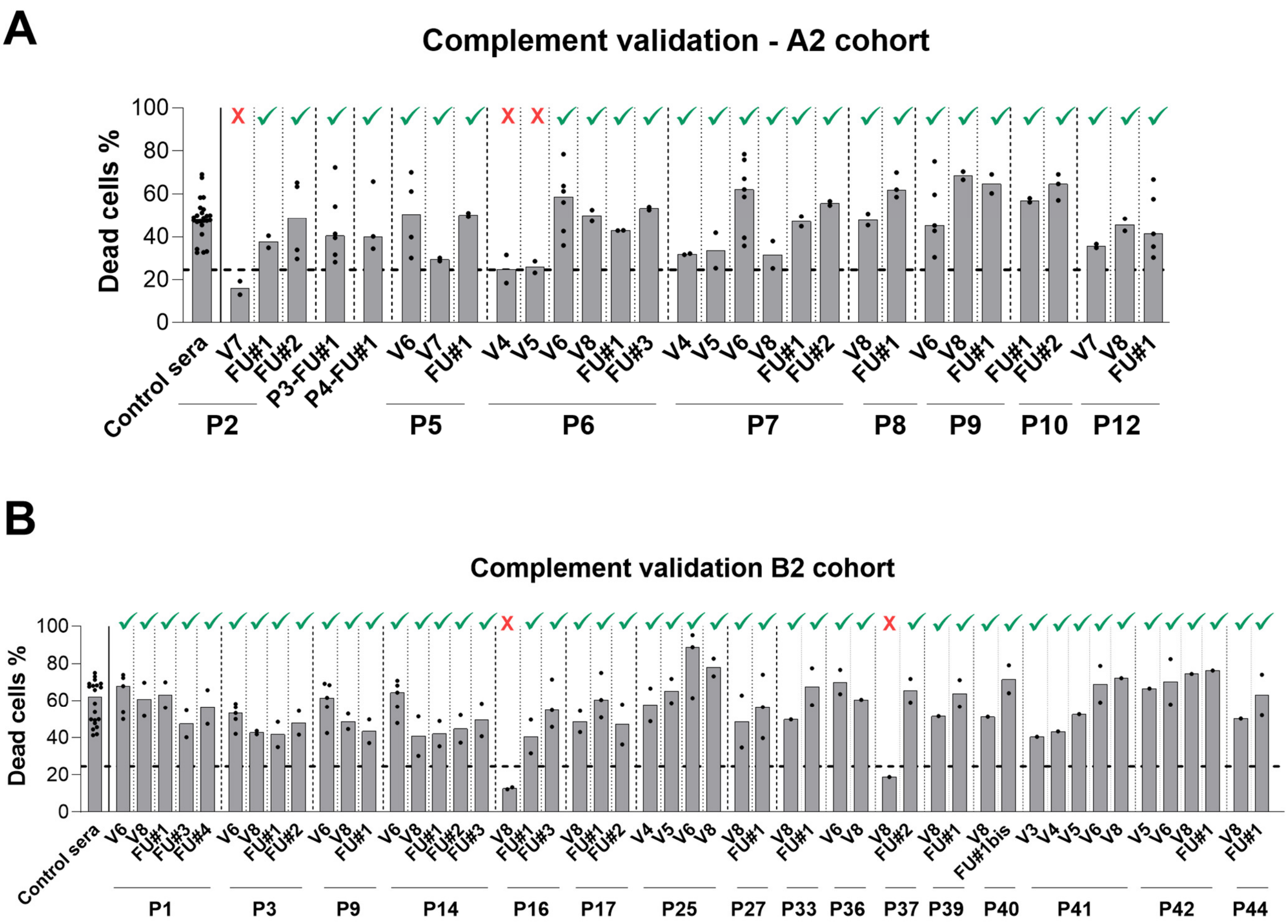

Figure S9. Patients' complement validation. The functionality of patients' complement was evaluated by measuring the capacity of the patient's untreated serum to induce CDC on control B cells (HD#01, HD#02 and HD#03) in the presence of control anti-HLA antibodies in large excess compared to patients' anti-HLA antibodies. The patients' sera from A2 (**A**) and B2 cohorts (**B**) were evaluated at different time points and compared to the CDC induced by control anti-HLA antibodies in the presence of human serum pool containing functional complement (control sera). The dotted lines represent the 24.5% positivity threshold for complement validation. A validated patients' complement is represented by a green tick, and a non-validated one by a red cross. The medians are shown. For control sera, N=20 in **A** and N=24 in **B**. For patients (P), 1-6 experiments were done. V=visit; FU=Follow-Up.

Figure S10

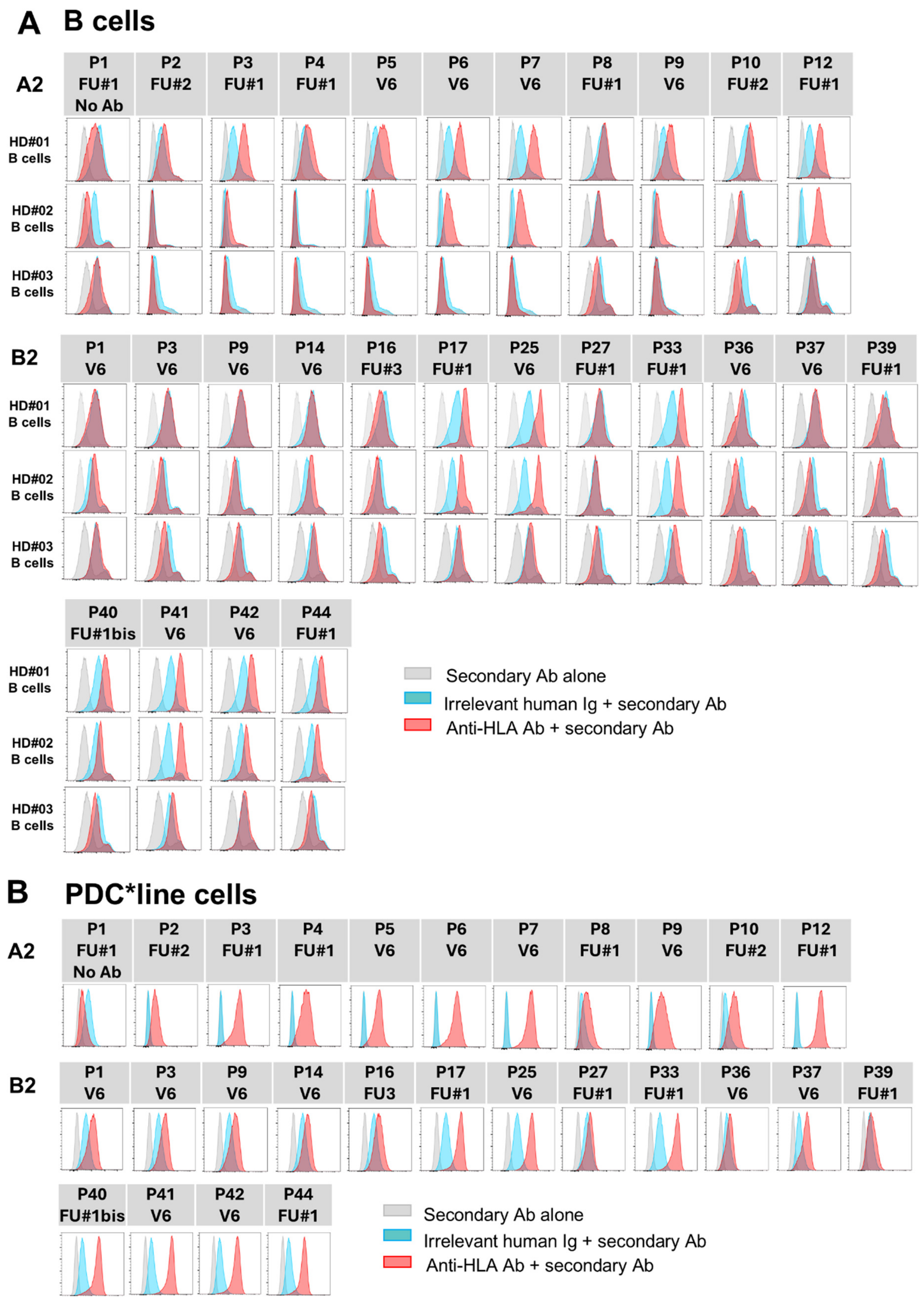

Figure S10. Recognition of PDC*line cells and control B cells by anti-HLA antibodies of Cohort A2 and B2 patients. Patients' sera were incubated with two allogeneic B cells (**A**, HD#01 and HD#02), unmatched B cells (**A**, HD#03) and PDC*line cells (**B**). The binding of antibodies was measured by labelling cells with fluorescent secondary anti-IgG antibodies. Non-specific binding

was evaluated replacing patients' sera by human serum pool providing irrelevant immunoglobulins (Irrelevant human Ig). Negative control was done labelling cells with secondary antibodies alone. The results are presented from left to right according to the patient (P) number. FU: Follow-up; V6: Visit 6.

Figure S11

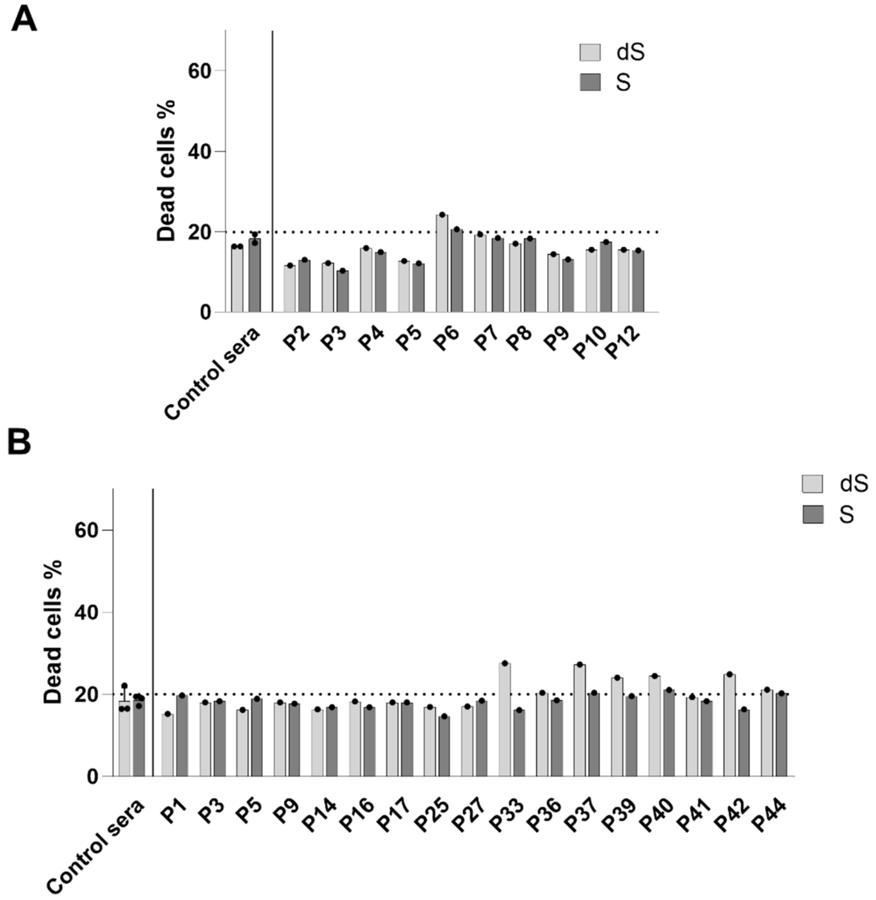

Figure S11. Complement-dependent cytotoxicity (CDC) activity of cohort A2 (A) and B2 (B) patient's sera against unmatched B cells (HD#03). The percentage of cell death is shown in untreated (S) and decomplemented serum (dS) conditions. Pools of sera from healthy donors were used as negative controls (Control sera). Patients B2-P17 and B2-P33 have anti-DPB1*04:01 antibodies greater than 3,000 of MFI. The means of 2 (**A**) or 3 (**B**) experiments +SD are shown for control sera. One experiment was done with patients' sera. The dotted lines represent the 20% positivity threshold.

Figure S12

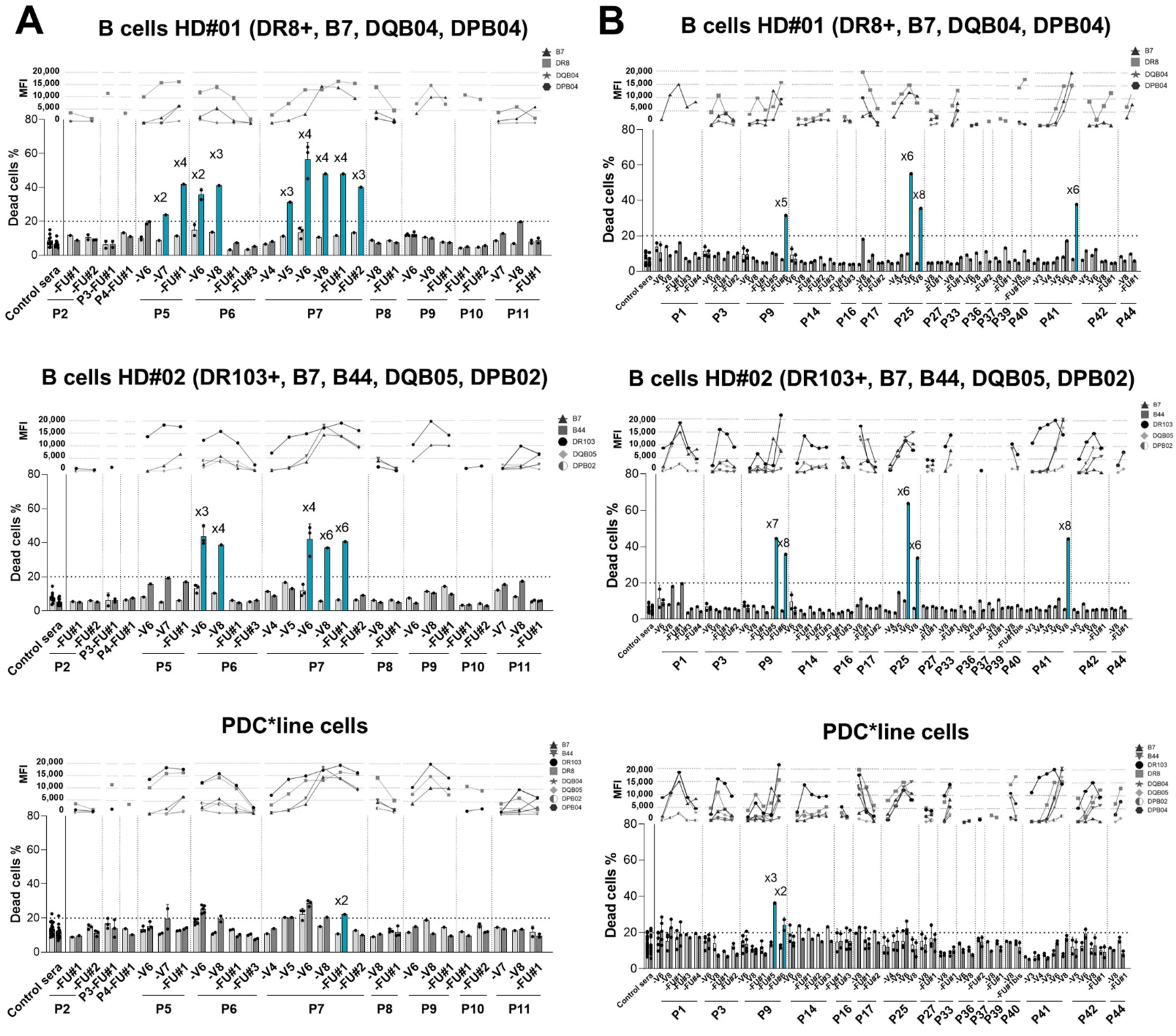

Figure S12. Kinetics of CDC activity according to anti-HLA levels. The sera of patients (P) from Cohort A2 (**A**) and B2 (**B**) were collected at different timepoints during the treatments (see supplementary Figure 1 for the trial timeline). Anti-HLA antibodies were measured in each serum with Luminex technology. The line graphs above bar plots present the mean fluorescence intensity (MFI) of detected anti-HLA antibodies. The levels of anti-HLA Class I (B7 and B44) and Class II (DR103, DR8, DQB04, DQB05, and DPB02), which are matching HLA typing of the indicated cell type are shown. In parallel, CDC activity of each serum was evaluated against primary B cells from two allogeneic healthy donors (HD#01 and HD#02) and PDC*line cells. Pools of sera from healthy donors were used as negative controls (Control sera). The bar plots show the percentage of cell death in untreated (S) and decomplemented serum (dS) conditions (means +SD; n=1-3 for patients; n=11-28). The horizontal dotted line indicates the 20% positivity threshold. The fold change of cytotoxicity between S and dS conditions is indicated above green bars when it is over the twofold positivity threshold.

Figure S13

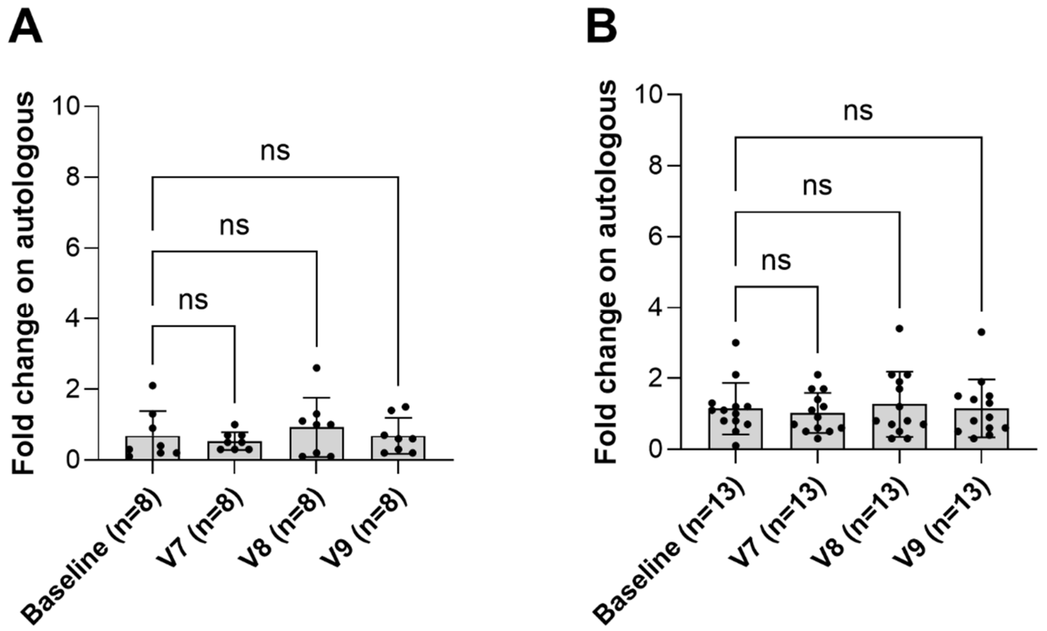

Figure S13. Cellular alloreactivity of patients with humoral responses in A2 (A) and B2 (B) cohorts. The allogeneic proliferation of PBMCs from anti-HLA positive patients was measured in a mixed leucocyte reaction assay at the indicated timepoints using 60Gy-irradiated PDC*line cells as stimulator cells and the patient's PBMCs as responder cells. The graphs show the fold change of the fluorescent signal measured in the condition with patients' PBMCs compared to the negative control autologous condition (healthy donors' PBMCs cocultured with the same irradiated PBMCs). Each circle represents the median of at least six replicates for each patient. The bars show the mean +SD of results from 8 (Cohorts A in A) and 13 (Cohorts B in B) patients. ns: not significant p-value evaluated with Friedman test.

Supplementary Tables

Table S1. Patient Demographics.

Cohorts	A1	A2	B1	B2
**Nb patients**	6	12	7	48
**Gender**				
Male, n (%)	5 (83)	10 (83)	4 (57)	27 (56)
Female, n (%)	1 (17)	2 (17)	3 (43)	21 (44)
**Age**				
years, median (range)	64.0 (40–71)	65.5 (50–71)	64.0 (39–78)	68.5 (50–83)
**Ethnicity, n (%)**				
Caucasian	4 (67)	8 (67)	6 (86)	47 (98)
Asian	0 (0)	0 (0)	1 (14)	0 (0)
Missing	2 (33)	4 (33)	0 (0)	1 (2)
**Performance status, n (%)**				
0	3 (50)	7 (58)	3 (43)	13 (27)
1	3 (50)	5 (42)	4 (57)	35 (73)
**PD-L1 expression**				
%, median	NA	NA	80	70
**Stage, n (%)**				
IIA	-	2 (17)	-	-
IIB	3 (50)	6 (50)	-	-
IIIA	3 (50)	4 (33)	-	-
IVA	-	-	3 (43)	19 (40)
IVB	-	-	4 (57)	29 (60)
**Histopathology subtype, n (%)**				
Squamous cell carcinoma	1 (17)	5 (42)	2 (29)	10 (21)
Adenocarcinoma	5 (83)	7 (58)	3 (43)	36 (75)
Other*	0	0	2 (29)	2 (29)
**Presence of anti-HLA at Baseline against other molecules than those expressed by PDC*line cells****				
n (%)	0 (0)	4 (33.3)	1 (14.3)	5 (10.4)

NA, not available; PD-L1, programmed death ligand 1; * B1: pleomorphic and large cells, not otherwise specified, B2: not otherwise specified for both. ** 50% of pre-immunized patients were female.

Table S2. HLA Typing of healthy donors (HD).

HD#01, HD#02, and HD#03 were used for Complement-Dependent Cytotoxicity assay and flow cytometry cross-match assay. HD#04, HD#05, HD#06, and HD#07 were used for Lymphocytotoxicity assay.

Donors/Alleles	A (1)	A (2)	B (1)	B (2)	C (1)	C (2)	DRB1 (1)	DRB1 (2)	DQB1 (1)	DQB1 (2)	DPB1 (1)	DPB1 (2)
**HD#01**	02:01	02:01	07:02	51:01	03:04	07:02	08:02	15:01	04:02	06:02	04:01	04:01
**HD#02**	02:01	03:01	07:02	44:03	07	15	01:03	11	03	05	02:01	04:01
**HD#03**	01:01	02:01	08:01	15:01	03:03	07:01	03:01	15:01	02:01	06:02	04:01	04:02

**HD#04**	02:01	02:01	07:02	15:01	03:03	07:02	13:01	15:01	06:02	06:03	02:01	04:01
**HD#05**	02:01	02:01	44:02	44:02	05:01	05:01	04:01	04:01	03:01	03:01	04:01	04:01
**HD#06**	01:03	03:01	08:01	47:01	06:02	07:01	01:03	03:01	02:01	03:01	04:02	04:02
**HD#07**	02:01	24:02	15:01	35:02	04:01	07:02	03:01	08:01	02:01	04:02	02:01	03:01

Table S3. C3d deposition and Lymphocytoxicity (LCT) results.

The C3d was measured using Lifecodes C3d, Lifecodes Single Antigen assays and Luminex technology. The LCT was performed according to Terasaki method with patients' sera and matched B cells (Table S2) in presence or absence of dithiothreitol (DTT) to determine IgM Abs implication.

	C3d positivity (>5 000)		LCT Class I and Class II		Isotype identification			
					IgM+/-IgG		IgM only	
Cohort	N	n (%)	N	n (%)	N	n (%)	N	n (%)
A2	10	6 (60)	10	9 (90)	10	7 (70)	9	3 (33.3)
B2	11	8 (72.7)	22	19 (86.4)	22	15 (68.2)	22	12 (54.5)*

N= number of tested patients
n= number of positive patients
%= frequence of positivity considering the number of tested patients
*: not significatively different from the result in Cohort A2 (chi2 test, GraphPad PRISM)
